# Regulation of CRISPR trans-cleavage activity by an overhanging activator

**DOI:** 10.1093/nar/gkaf117

**Published:** 2025-02-24

**Authors:** Na Yin, Hongyan Yu, Li Zhang, Fei Luo, Weitao Wang, Xiaole Han, Yu He, Yiqi Zhang, You Wu, Jiu Pu, Tong Feng, Gang Yang, Tingmei Chen, Guoming Xie

**Affiliations:** Key Laboratory of Clinical Laboratory Diagnostics (Chinese Ministry of Education), College of Laboratory Medicine, Chongqing Medical University, Chongqing 400016, P.R. China; Key Laboratory of Clinical Laboratory Diagnostics (Chinese Ministry of Education), College of Laboratory Medicine, Chongqing Medical University, Chongqing 400016, P.R. China; Key Laboratory of Clinical Laboratory Diagnostics (Chinese Ministry of Education), College of Laboratory Medicine, Chongqing Medical University, Chongqing 400016, P.R. China; Key Laboratory of Clinical Laboratory Diagnostics (Chinese Ministry of Education), College of Laboratory Medicine, Chongqing Medical University, Chongqing 400016, P.R. China; Key Laboratory of Clinical Laboratory Diagnostics (Chinese Ministry of Education), College of Laboratory Medicine, Chongqing Medical University, Chongqing 400016, P.R. China; Key Laboratory of Clinical Laboratory Diagnostics (Chinese Ministry of Education), College of Laboratory Medicine, Chongqing Medical University, Chongqing 400016, P.R. China; Key Laboratory of Clinical Laboratory Diagnostics (Chinese Ministry of Education), College of Laboratory Medicine, Chongqing Medical University, Chongqing 400016, P.R. China; Key Laboratory of Clinical Laboratory Diagnostics (Chinese Ministry of Education), College of Laboratory Medicine, Chongqing Medical University, Chongqing 400016, P.R. China; Key Laboratory of Clinical Laboratory Diagnostics (Chinese Ministry of Education), College of Laboratory Medicine, Chongqing Medical University, Chongqing 400016, P.R. China; Key Laboratory of Clinical Laboratory Diagnostics (Chinese Ministry of Education), College of Laboratory Medicine, Chongqing Medical University, Chongqing 400016, P.R. China; Key Laboratory of Clinical Laboratory Diagnostics (Chinese Ministry of Education), College of Laboratory Medicine, Chongqing Medical University, Chongqing 400016, P.R. China; Department of Neurosurgery, The First Affiliated Hospital of Chongqing Medical University, Chongqing 400016, P.R. China; Key Laboratory of Clinical Laboratory Diagnostics (Chinese Ministry of Education), College of Laboratory Medicine, Chongqing Medical University, Chongqing 400016, P.R. China; Key Laboratory of Clinical Laboratory Diagnostics (Chinese Ministry of Education), College of Laboratory Medicine, Chongqing Medical University, Chongqing 400016, P.R. China; Western Institute of Digital-Intelligent Medicine, Chongqing 401329, P.R. China

## Abstract

The clustered regularly interspaced short palindromic repeats (CRISPR)/Cas12a system exhibits extraordinary capability in the field of molecular diagnosis and biosensing, attributed to its *trans*-cleavage ability. The precise modulation of performance has emerged as a significant challenge in advancing CRISPR technology to the next stage of development. Herein, we reported a CRISPR/Cas12a regulation strategy based on an overhanging activator. The presence of overhanging domains in activators creates steric hindrances that have a substantial impact on the *trans*-cleavage activity and activation timing of Cas12a. The *trans*-cleavage activity of Cas12a can be finely tuned by adjusting the position, length, and complementarity of the overhanging domains. Moreover, specific structures exhibit characteristics of automatic delayed activation. The presence of overhanging domains enables precise and timely activation of Cas12a, facilitating multifunctional applications. This system effectively accomplishes dynamic regulation, programmable release of cargo, logical operations, and multi-enzyme detection. The flexibility and versatility of this simple and powerful CRISPR regulatory strategy will pave the way for expanded applications of CRISPR/Cas in biotechnology, bioengineering, and biomedicine.

## Graphical Abstract

**Graphical Abstract:**
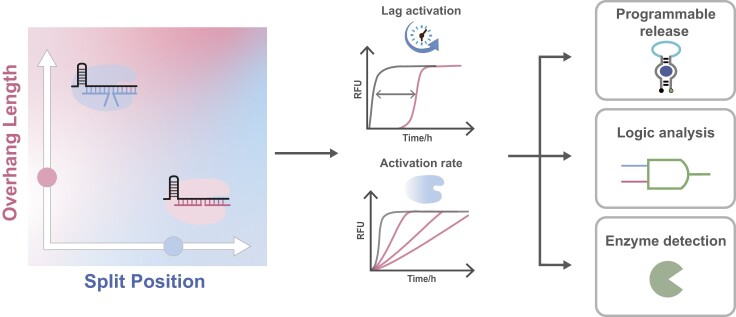

## Introduction

The clustered regularly interspaced short palindromic repeats (CRISPR) and CRISPR-associated (Cas) proteins are derived from the acquired immune system of prokaryotes, enabling them to defend against the invasion of exogenous genetic factors in bacteriophages or plasmids [1, 2]. In prokaryotes, there exist several naturally occurring CRISPR/Cas systems, with Cas12a belonging to the class II type V category as a RNA-guided DNA endonuclease [3, 4]. RNA-guided DNA recognition initiated by identifying the T-rich protospacer-adjacent motif (PAM) on target double-stranded DNA (dsDNA), allowing the unwinding of dsDNA and Watson–Crick base pairing between the target strand (TS) and CRISPR RNA (crRNA) [5]. Following this, Cas12a employs a single RuvC domain to cut the TS and nontarget strand (NTS) in sequential mechanism. Moreover, activated Cas12a are capable of nonspecific cleavage of single-stranded DNA (ssDNA), known as *trans*-cleavage. Given these attributes of Cas12a, it has found extensive applications in various domains of molecular biology, including genome editing, molecular diagnostics, transcriptional regulation, gene therapy, intracellular imaging, and molecular biochemical circuits [6–11].

However, with the continuous expansion of applications, the inherent properties of CRISPR/Cas are no longer sufficient to meet the diverse needs of more and more applications. The precise regulation of Cas12a *trans*-cleavage activity is expected to further promote the understanding of the mechanism and application of Cas12a system. The crRNA plays a pivotal role in the recognition function and serves as a core component of the CRISPR/Cas system. Extensive research efforts focus on the modification of crRNA, including circular crRNA [12], photo controlled crRNA activation [13], G-quadruplex guided RNA (gRNA)[14, 15], strand displacement gRNA [16], methylated gRNA [17], engineered crRNA [18], and tandem crRNA [19] to achieve CRISPR/Cas signal switching and activity regulation. However, emerging challenges have impeded its advancement to the next stage, such as the complexity for elaborately design crRNA and the potential biological hazards caused by chemical modification methods. The programmatic modification of activators beyond crRNA shows significant promise for development and has recently attracted the attention of researchers [20–23]. The majority of these regulation methods aim to enhance or weaken the *trans*-cleavage activity of Cas12. In living organisms, enzyme activity and activation timing are delicately regulated by a variety of complex mechanisms to ensure timely metabolic processes and accurate regulation, preventing premature or excessive enzyme activation and thereby avoiding metabolic disorders in cells. Therefore, it is attractive to develop a flexible regulatory mechanism that can automatically achieve delayed activation of the CRISPR/Cas12a system.

Many biological processes, including the regulation of metabolism, signal transduction, and physiological reactions, exhibit various flexible mechanisms that ensure enzyme activity is precisely activated under the appropriate conditions and at the right time. This optimization enhances energy utilization, maintains physiological balance, improves environmental adaptability, and facilitates signal amplification [24, 25]. DNA nanotechnology, with its programmable modules, fine-tuned dynamics, and predictable structures, is frequently conjugated with enzymes and provides an easy-to-use toolbox for constructing synthetic molecular systems with time-control capabilities [26–31]. In previous work, we demonstrated the regulatory ability of CRISPR/Cas through the utilization of toehold mediated strand displacement to activate crRNA [32, 33] and found that elongated ssDNA activators had an impact on *trans*-cleavage kinetics. It remains unclear if the pre-designed activator structure can modulate the activation mechanism of Cas12a, enabling precise activation at the appropriate time and under specific conditions.

Inspired by the above, we find that overhanging domains (the extended domains at the end of the activator) can regulate the *trans*-cleavage activity of Cas12a and specific structures exhibit characteristics of automatic delayed activation. We initially investigated the effect of split activators on the *trans*-cleavage activity of CRISPR–Cas12a, followed by the development of overhanging activators. Modulating the position, length, and complementarity of the overhanging domains enables the precise regulation of the *trans*-cleavage activity and activation timing of Cas12a. This regulatory approach is applicable in both ssDNA and dsDNA activation modes, offering controllability, dynamism, and flexibility. We demonstrate the robust potential of this activation strategy and further realize more intriguing and practical functionalities: programmable release of ATP through postponed activation of Cas12a subsequent to aptamer-mediated ATP loading; dynamic activation and inhibition of Cas12a *trans*-cleavage activity based on strand displacement reaction; logical signal analysis of influenza virus coupled with strand displacement amplification (SDA); and universal detection system for APE1, uracil-DNA glycosylase (UDG), and ribonuclease (RNase) H. This system holds significant promise in molecular diagnostics, drug delivery, and bioengineering applications.

## Materials and methods

### Materials

Oligonucleotides were purchased from Beijing Genomics Institution (Beijing, China), Tsingke Biotechnology Co. Ltd (Beijing, China), and Sangon Biotech Co. Ltd (Shanghai, China). All DNA and RNA sequences are listed in Supplementary Table S1. Lba Cas12a (Cpf1, 20 μM), rCutSmart Buffer, purinic/apyrimidinic endonuclease 1, UDG, RNase H, RNase H Reaction Buffer, BstNI, NEBuffer, EXO I, Nt.BsmAI, ATP, Bst DNA polymerase, ThermoPol, and Nt.BstNBI were bought from New England Biolabs (Beijing, China). dNTP (100 mM) and dithiothreitol (DTT) were bought from Sangon Biotech. N,N,N′,N′-tetramethylethylenediamine and 30% acrylamide/bis solution were provided by Sigma–Aldrich (St Louis, MO, USA). DNA loading buffer (6×) and Gel Red nucleic acid dye were ordered from TaKaRa Biotech (Dalian, China).

### Instruments

Time-based fluorescence data were acquired using a RotorGene 6000 instrument (Corbett Research, Mortlake, Australia). The temperature was set to 37°C, and gain was set to default. All fluorescent signals were monitored under the yellow or green channel. Assembly of oligonucleotides was achieved by annealing in a polymerase chain reaction instrument (CFX96, Bio-Rad, USA). The procedure was performed at 95°C for 5 min and then the temperature was decreased from 95°C to 12°C at the rate of 0.1°C/s. Gel images were obtained on an electrophoresis apparatus (DYY-6C, LIUYI, China) and imaging system (Bio-Rad Laboratories, USA).

### Model of *trans*-nuclease activation reactions

To describe this process mathematically, we assume a three-step kinetic model for target-induced enzyme activation: the activator T binds reversibly to the ribonucleoprotein RNP to form an enzyme–activator complex RNPT; the enzyme–activator complex RNPT is converted irreversibly to the activated enzyme RNPT*; and the activated enzyme RNPT* catalyzes the conversion of the substrate reporter to the product P, which may be depicted by equation (1):


(1) \begin{eqnarray*} &&{\mathrm{RNP\;}} + {\mathrm{\;T\;}}_{\mathop \leftarrow \limits_{{\mathrm{\;}}{{\mathit{k}}_{{\mathrm{off}}}}{\mathrm{\;}}} }^{\mathop \to \limits^{{\mathrm{\;}}{{\mathit{k}}_{{\mathrm{on}}}}{\mathrm{\;}}} }{\mathrm{\;RNPT\;}}\mathop \to \limits^{{\mathrm{\;}}{{\mathit{k}}_{{\mathrm{act}}}}{\mathrm{\;}}} {\mathrm{\;RNP}}{{\mathrm{T}}^{\mathrm{*}}}\nonumber\\&& \quad+ {\mathrm{\;Reporter\;}}\mathop \to \limits^{{\mathrm{\;}}{{\mathit{k}}_{{\mathrm{cat\;}}}}} {\mathrm{\;RNP}}{{\mathrm{T}}^{\mathrm{*}}}{\mathrm{\;}} + {\mathrm{\;P}},\end{eqnarray*}

where *k*_on_ is the association rate constant of RNP and T, *k*_off_ is dissociation rate constant of RNPT, the intrinsic affinity of RNP for target can be calculated as: *K*_d_= *k*_on_/*k*_off_, *k*_act_ is a rate for RNP activation, manifested as a lag in the time course, and *k*_cat_ is the turnover number of activated CRISPR.

The steps of activator binding and enzyme activation can be mathematically modeled by the ordinary differential equations:


(2) \begin{eqnarray*} &&{{\mathrm{d}}\left[ {{\mathrm{RNP}}} \right]/{\mathrm{d}\mathit{t}} = - {{\mathit{k}}_{{\mathrm{on}}}}\left[ {{\mathrm{RNP}}} \right]\left[ {\mathrm{T}} \right] + {{\mathit{k}}_{{\mathrm{off}}}}\left[ {{\mathrm{RNPT}}} \right]}\nonumber\\&& {{\mathrm{d}}\left[ {\mathrm{T}} \right]/{\mathrm{d}\mathit{t}} = - {{\mathit{k}}_{{\mathrm{on}}}}\left[ {{\mathrm{RNP}}} \right]\left[ {\mathrm{T}} \right] + {{\mathit{k}}_{{\mathrm{off}}}}\left[ {{\mathrm{RNPT}}} \right]}\nonumber\\ && {{\mathrm{d}}\left[ {{\mathrm{RNPT}}} \right]/{\mathrm{d}\mathit{t}} = {{\mathit{k}}_{{\mathrm{on}}}}\left[ {{\mathrm{RNP}}} \right]\left[ {\mathrm{T}} \right] - {{\mathit{k}}_{{\mathrm{off}}}}\left[ {{\mathrm{RNPT}}} \right] - {{\mathit{k}}_{{\mathrm{act}}}}\left[ {{\mathrm{RNPT}}} \right]}\nonumber\\&& {{\mathrm{d}}\left[ {{\mathrm{RNPT}}^{\mathrm{*}}} \right]/{\mathrm{d}\mathit{t}} = {{\mathit{k}}_{{\mathrm{act}}}}\left[ {{\mathrm{RNPT}}} \right]}. \end{eqnarray*}

The activated enzyme RNPT* binds to the substrate reporter, and catalyzes its conversion to the product P, which follows Michaelis–Menten kinetics:


(3) \begin{eqnarray*}\frac{{{\mathrm{d}}\left[ {\mathrm{P}} \right]}}{{{\mathrm{d}\mathit{t}}}}{\mathrm{\;}} = - \frac{{{\mathrm{d}}\left[ {{\mathrm{Reporter}}} \right]}}{{{\mathrm{d}\mathit{t}}}} = {\mathit{\;v}} = \frac{{{\mathrm{\;}}{{\mathit{k}}_{{\mathrm{cat}}}}\left[ {{\mathrm{RNP}}{{\mathrm{T}}^{\mathrm{*}}}} \right]\left[ {{\mathrm{Reporter}}} \right]}}{{{K_{\mathrm{M}}} + \left[ {{\mathrm{Reporter}}} \right]}},\nonumber\\\end{eqnarray*}

where *K*_M_ is the Michaelis–Menten constant.

For processes displaying explicit lagged kinetics, we used a delay differential equation model to rigorously simplify multiple steps (RNP activation) into a single step with delay: d[RNPT*]/d*t* = *k*_act_[RNPT](*t* − τ), where [RNPT](*t* − τ) represents the concentration of RNPT at a time τ units in the past, making the effect of RNPT on the current rate of change of RNPT* lagged by a characteristic time τ.

All fittings were performed with the MATLAB function *lsqcurvefit*, which solves nonlinear least-squares problems by minimizing the Euclidian norm of the residuals. The value of *k*_act_, *k*_cat_, and *K*_M_ were fitted using the individual trajectories of system with a simplified ODE model consisting of equations ((2)) and ((3)). The characteristic time τ was fitted using nonlinear delay differential equations.

### Preparation of complexes

Cas12a and crRNA complexes: Cas12a and crRNA were mixed at a 1:1 ratio and pre-incubated at 37°C for 30 min to promote the ribonucleoprotein complex (RNP, 400 nM) formation. dsDNA complexes: TS and NTS strands are at a 1:1 ratio annealed to form dsDNA complexes (2.5 μM).

### Programmable ATP release and dynamic regulation

For length regulation, a volume of 20-μl reaction solution was prepared containing 1 mM ATP, 250 nM ATP aptamer, 40 nM RNP, 1× CutSmart Buffer, and 250 nM various length of activators. For concentration regulation, a volume of 20-μl reaction solution was prepared containing 1mM ATP, 250 nM ATP aptamer, 80 nM RNP, 1× CutSmart Buffer, and various concentration of activators. For dynamic regulation of crRNA activation, the reaction mixture was prepared containing 100 nM RNP, 1× CutSmart Buffer, 250 nM Cas-reporter, and 1 mM DTT. The fluorescence was then recorded every 1 min for 4 min. First, activator was added to the reaction solution to a final concentration of 10 nM. After 4 min, inhibitor was then added at a final concentration of 20 nM. After next 4 min, activator was then added at a final concentration of 50 nM. Again after 4 min, inhibitor was then added at a final concentration of 120 nM. Finally, after next 4 min, activator was then added at a final concentration of 250 nM.

### Logic gate signal analysis

The initial stage involves BstNI to precisely cleave DNA in RNA/DNA heteroduplexes to transform Influenza A/B RNA into short DNA. A total of 20 μl of mixture, containing 8 μl of Influenza A/B RNA solution (5 μM), 8 μl of converter DNA A/B (10 μM), 0.8 μl of diethyl pyrocarbonate-treated water, 1.5 μl of NEBuffer 3.1 (10×), and 1.7 μl of BstNI (10 U/μl), was incubated at 55°C for 10 min. A volume of 20-μl SDA amplification reaction solution was prepared containing 100 nM template, 1× ThermoPol, 1× NEBuffer, 1 U Nt.BstNBI, 1 U Bst DNA polymerase, 1 mM dNTPs, and RNase-free water. Then, 10 μl of the transformation product was mixed with the SDA reaction solution, followed by incubation at 55°C for 30 min. Finally, the reaction was terminated by subjecting to 85°C for 10 min. Then a volume of 20-μl CRISPR/Cas12a reaction solution was prepared containing 10 μl of amplification product, 50 nM preloaded activators, 40 nM RNP, 1× CutSmart Buffer, 250 nM Cas-reporter, 1 mM DTT, and deionized water.

### Universal CRISPR/Cas12a system for multi-enzyme assays

AP site strand, dU site strand, DNA–RNA composite strand, respectively, annealed with complementary strand and NTS to form dsDNA (2.5 μM) complexes. In order to detect APE1, a volume of 10-μl enzyme digestion reaction solution containing AP site-complex (500 nM), 1× NEBuffer, 1 μl of APE1 (at various concentrations), and deionized water was prepared. After 35 min incubation at 37°C, the above solution was heated at 65°C for 20 min to make APE1 inactive. Then, 250nM split activator, 40 nM RNP, 1× CutSmart Buffer, 250 nM Cas-reporter, 1 mM DTT, and deionized water were added to make the total volume 20 μl. The detection methods of UDG and RNase H were similar to APE1. To study the selectivity of the method, RNase H, UDG, Nt.BsmAI, and Exo I were selected as the potential interfering enzymes to compare with APE1.

## Results

### The effect of activators with overhanging domains on *trans*-cleavage activity in single-domained activation mode

**Figure 1.**
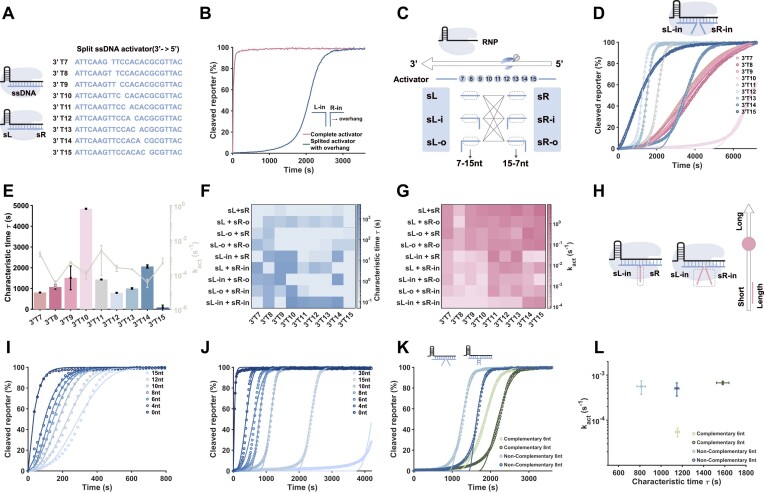
(**A**) Design of activator breaks. (**B**) The fluorescence kinetics of complete activator and overhanging domains activator. (**C**) The positional combination pattern of split activator with overhanging domains. (**D**) The fluorescence kinetics of the overhanging domain inside of sL and sR and (**E**) its characteristic time and the rate of activation when position changes. Characteristic time (**F**) and the rate of activation (**G**) of nine combinations when breakpoint position changes. (**H**) Scheme for regulating the length. Overhanging domains are all set up inside. (**I**) Changes in the length of a single overhanging domain allow for a short range of regulation. (**J**) Changes in the length of the two overhanging chains allow for a long range of regulation. (**K**) Regulation of activation by complementarity. (**L**) Characteristic time and the rate of activation of complementary and noncomplementary overhanging domains. The concentration of RNP is 40 nM, the activator concentration is 250 nM, and the reporter is 250 nM.

We first investigated the effect of the overhanging strand on the *trans*-cleavage activity of Cas12a. By altering the breakpoint position 3′T*x* (3′T*x* indicates position *x* base from 3′ end of complete activator T, *x* is ranging from 7 to 15), ssDNA is programmatically split into two parts (sL and sR) (Fig. 1A). As the breakpoint position approaches the 3′ end of the activator, the apparent *trans*-cleavage activity of Cas12a decreases by approximately one order of magnitude. The 3′T8 activator exhibits an anomalous reduction in apparent *trans*-cleavage activity, possibly due to its secondary structure hindering Cas12a activation. Furthermore, by adding 10-nt overhangs to both inner ends of the split activator, we observed an obvious lag phase in Cas12a *trans*-cleavage (Fig. 1B).

Next, we explored the mechanisms underlying the occurrence of lagged activation. The overhanging domain is designed at different positions of the activator, which can be situated either inside or outside the breakpoint, labeled as sL-i (inside), sL-o (outside), sR-i (inside), and sR-o (outside), resulting in a total of nine combinations. Additionally, the overhanging domain can be encoded by varying the breakpoint. Each combination sets the breakpoint at nine distinct positions(T7–T15), with the overhang length fixed at 10 nt (Fig. 1C). When a single overhanging domain is positioned on the outside of the split activator (sL-o + sR or sL + sR-o), the overhanging domain has minimal effect on *trans*-cleavage activity of Cas12a, with kinetics similar to those of split ssDNA (Supplementary Figs S1–S2). In contrast, positioning a single overhanging domain on the inner side of the split activator (sL-i + sR or sL + sR-i) led to a substantial impact on *trans*-cleavage kinetics at specific positions (Supplementary Fig. S3). Lagged activation was observed at 3′T9 and 3′T10, with characteristic times exceeding 10 min. We further increased the number of overhang domain to two and found that the outer overhanging domain had little effect on the *trans*-cleavage activity of Cas12a (Supplementary Fig. S4). When one of the overhangs was shifted from external to internal, at the same split position, the characteristic time of the lag phase at the same split position was further prolonged compared with that observed with a single internal overhanging domain. This indicates that the *trans*-cleavage activity is dominated by the internal overhang (Supplementary Fig. S5). When both overhanging domains were located inside (sL-i + sR-i), the *trans*-cleavage kinetics were significantly affected at all split positions. The lag phase characteristic time was notably longer when the breakpoint was centrally located, reaching up to nearly 5000 s (Fig. 1D and E). The observed effect may arise from spatial steric hindrance caused by the overhang at the middle position, which interferes with the binding of Cas12a, crRNA and the activator, thereby extending the activation time for Cas12a *trans*-cleavage activity. These findings demonstrate that the internal overhanging domain plays a dominant role in regulating *trans*-cleavage activity. Notably, as the overhanging domain approaches the middle position, the *trans*-cleavage rate decreases, and lag phase becomes more pronounced (Fig. 1F and G).

To investigate the effect of overhanging length on Cas12a activity, the overhang was designed to be positioned inside sL and sR, with the breakpoint located at 3′T11 or 3′T12 (Fig. 1H). Fig. 1I demonstrates that altering the length of a single overhanging domain enables precise regulation of Cas12a activity with characteristic timescales on the order of tens of seconds, varying the lengths of two overhanging domains expands the characteristic timescales to thousands of seconds (Fig. 1J and Supplementary Fig. S7). Polyacrylamide gel electrophoresis (PAGE) was also employed to verify the lagged activation of Cas12a (Supplementary Fig. S7). Compared with regulating Cas12a *trans*-cleavage activity by adjusting the split position of activators, tuning the overhang length offers greater precision in controlling the characteristic time and enhanced programmability. This approach provides an effective means for the precise regulation of Cas12a *trans*-cleavage activity. In addition, we observed that lagged activation was more pronounced when the noncomplementary overhang became complementary, which may be attributed to the increased steric hindrance formed as a result of the complementation (Fig. 1K and L).

Regarding the lagged activation phenomenon, we speculated that the presence of the overhanging domain interferes of the activators and RNP, thereby increasing the activation time of Cas12a. To verify this, we compared the kinetics of *trans*-cleavage activity between pre-incubated and unincubated RNP and activators. The results indicated that, in contrast to the lagged activation pattern observed in the absence of incubation, the fluorescence signal was generated immediately after incubation, although at a reduced *trans*-cleavage rate (Supplementary Fig. S8A). The loosed *trans*-cleavage rate may be attributed to the *cis-*cleavage activity of Cas12a reducing the number of available activators. Extending the incubation time to ∼2 h further supported this observation (Supplementary Fig. S8B). Additionally, we pre-incubated crRNA with the activator for 1 h before adding the reporter and Cas12a for fluorescence monitoring (Supplementary Fig. S8C). The persistence of lag phase in the kinetic suggests that the delayed activation is not governed by the binding dynamics between the crRNA and the activators. The lagged activation phenomenon persists even after modifying the crRNA sequence (Supplementary Fig. S9). The observed different kinetics between distinct crRNA variants may be attributable to variations in their primary sequence or secondary structural characteristics.

### The effect of activators with overhanging domains on *trans*-cleavage activity in double-domained activation mode

**Figure 2.**
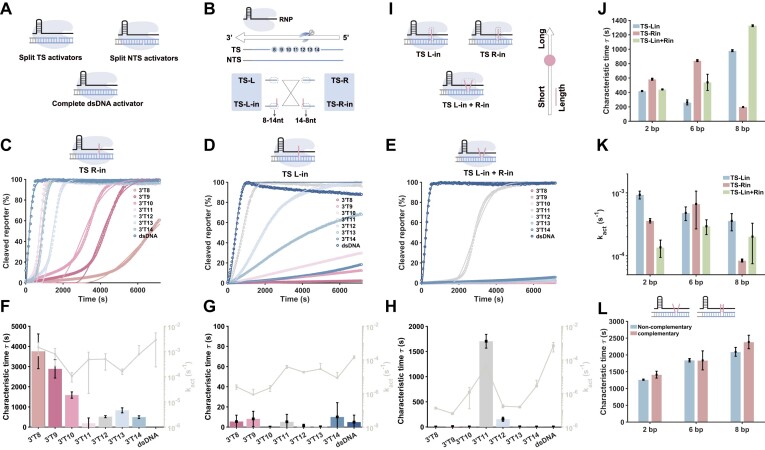
(**A**) Split dsDNA activator. (**B**) Scheme of overhanging domain on TS. (**C**) The fluorescence kinetics of the overhanging domain located inside of TS R when its position changes. (**D**) The fluorescence kinetics of the overhanging domain located inside of TS L when its position changes. (**E**) The fluorescence kinetics of the overhanging domain located inside of TS L and TS R when its position changes. (**F**) Characteristic time and the rate of activation of the overhanging domain located inside of TS R when its position changes. (**G**) Characteristic time and the rate of activation of the overhanging domain located inside of TS L when its position changes. (**H**) Characteristic time and the rate of activation of the overhanging domain located inside of TS L and TS R when its position changes. (**I**) Scheme for changing the length. (**J**) Characteristic time of the overhanging domain located inside of TS. (**K**) The rate of the overhanging domain located inside of TS. (**L**) Characteristic time of the double overhanging domains on TS when complementary and noncomplementary. The concentration of RNP is 40 nM, the activator concentration is 250 nM, and the reporter is 250 nM.

Similarly, we investigated whether dsDNA activators with overhanging domain could also regulate the *trans*-cleavage activity of Cas12a. Breakpoints were strategically designed on the NTS and TS, respectively (Fig. 2A). DsDNA activator was programmed by varying the cleavage position of 3′ T*x* (where 3′ represents the untruncated 3′ end of the unsplit TS, and *x* ranges from 8 to 14 bases, excluding the PAM nucleotides). The results demonstrated that the breakage of the NTS or TS led to a slight increase in the apparent rate of *trans*-cleavage, and changes in the breakpoint position within the TS were more sensitive, likely due to the presence of breakpoints facilitating crRNA recognition of the TS (Supplementary Fig. S10).

Next, we added the overhanging domain to split TS or NTS. The results demonstrated that incorporating the overhanging domain into the NTS and altering its position had minimal impact on the *trans*-cleavage activity of Cas12a (Supplementary Figs S11–S12). Then, we positioned an overhanging domain on the TS and varied its location (Fig. 2B). When a single overhanging domain was present in TS-R-in, the characteristic time of the lag phase was longer compared with the single-stranded activation mode, ranging from 100 to 4000s (Fig. 2C and F). When a single overhanging domain was present in TS-L-in, the characteristic time of the lag phase was not prominent; instead, the overall activation rate decreased, which differed from the phenomenon observed in the single-stranded activation mode (Fig. 2D and G). With two overhanging domains, breakpoints at 3′T11 exhibited a characteristic lag phase time of 1700 s, while Cas12a *trans*-cleavage activity at other positions was severely inhibited (Fig. 2E and H).

We further adjusted the length of the overhanging domain (Fig. 2I). Altering the length of the overhanging domains on NTS had minimal effect on the *trans*-cleavage activity of Cas12a (Supplementary Fig. S13). Similar to ssDNA activation, modifying the length of overhanging domains on the TS allowed for fine-tuning of the characteristic lag phase time or enabled broader regulation (Fig. 2J–K and Supplementary Fig. S14). When the overhang domains on the TS or NTS changes from noncomplementary to complementary, a delayed activation of Cas12a was also observed, with the characteristic time increasing as the complementary length increased (Fig. 2L and Supplementary Figs S15 and S16). In summary, the overhanging domains in the dsDNA activation mode can also modulate the *trans*-cleavage kinetics of Cas12a.

### Programmable ATP release and dynamic regulation

Nucleic acids (DNA and RNA) have become ideal components for nanotechnology, driven by their high programmability, predictable thermodynamics, and low synthesis costs. These features have spurred the development of nucleic acid self-assembly, nanomachines, and materials [34–38].

**Figure 3.**
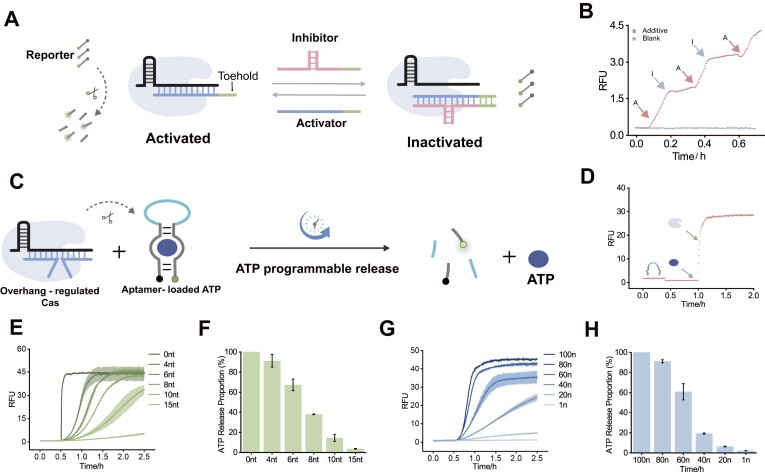
(**A**) Based on the strand displacement reaction, the alternating addition of activators and inhibitors with overhanging domains enables dynamic signaling regulation. (**B**) Fluorescence plot of dynamic regulation. The concentration of RNP is 100 nM, the initial activator is 10 nM, and the reporter is 250 nM. (**C**) The length or concentration of the overhanging activators can alter the effect of activation for programmable release of ATP. (**D**) Validation of ATP and aptamer binding and release. (**E**) The effect of length adjustment on ATP release. The activator concentration was fixed at 250 nM, RNP is 40 nM, and ATP aptamer is 250 nM. (**F**) The relative ratio of ATP release with different length. The 0 nt was defined as 100%, and the release rates at various lengths were calculated at 45 min. (**G**) The effect of concentration adjustment on ATP release. Overhanging domain was fixed at 4 nt. The concentration of RNP is 80 nM and ATP aptamer is 250 nM. (**H**) The relative ratio of ATP release with different concentration. The release rate at a concentration of 100 nM was defined as 100%, and the rates at different concentrations were calculated after 45 min.

A resettable network was designed based on strand displacement reactions and overhang domains. As illustrated in Fig. 3A, the activator complements the crRNA, activating the *trans*-cleavage activity of Cas12a and generating a fluorescent signal. Subsequently, an inhibitor (I) with an overhanging domain is introduced to the system. The addition of I binds to the toehold region of the activator (A), triggering strand displacement reaction that displaces A from the crRNA. On the one hand, A no longer binds to crRNA, and on the other hand the steric hindrance caused by the overhang inhibits the *trans*-cleavage activity of Cas12a, leading to a plateau in the fluorescence signal. The alternating addition of A and I facilitates dynamic regulation of Cas12a activity (Fig. 3B).

The controlled sequential release of various drugs, including soluble protein factors and small molecules, offers the potential to rectify abnormal biological events by enabling spatiotemporal control over local therapeutic levels [39, 40]. Here, we demonstrate the dynamic release of ATP as an example, successfully utilizing the CRISPR/Cas12a system with delayed activation effects for the controlled programmable release of ATP. As shown in Fig. 3C, we label the 3′ end of the ATP aptamer with a fluorescent group and the 5′ end with a quenching group. Upon binding of ATP to the aptamer, the distance between the two groups is reduced, leading to fluorescence quenching. When CRISPR/Cas12a is introduced, it cleaves the aptamer, releasing ATP and restoring fluorescence. We first validated that the aptamer can effectively load and release ATP. As shown in Fig. 3D, during the continuous reaction process, adding ATP to the aptamer causes a decrease in the fluorescence signal, consistent with ATP binding. Upon the addition of CRISPR/Cas12a, the aptamer is cleaved, resulting in an increase in fluorescence. We measured the fluorescence curves for conditions with no ATP, ATP addition, and ATP release (Supplementary Fig. S17). Subsequently, the length and concentration of the overhanging domain were adjusted in order to regulate the release of ATP. The results revealed that increasing the overhang length led to a reduction in ATP release (Fig. 3E). The ATP release at an overhang length of 0 nt was defined as 100%, and the release rates at various lengths were calculated at 45 min (Fig. 3F). When the overhang length was fixed at 4 nt, varying the concentration demonstrated that higher activator concentrations resulted in increased ATP release (Fig. 3G). The release rate at a concentration of 100 nM was defined as 100%, and the rates at different concentrations were calculated after 45 min (Fig. 3H). Therefore, this delayed ATP release system enables programmable control through the manipulation of length and concentration.

### Logic gate construction and influenza virus analysis

Boolean logic can be applied to various types of information represented by 0 (NO) and 1 (YES), making it widely used for qualitative analysis. Over the past decade, the stability, accessibility, and operability of DNA have led to significant research into the design and application of DNA logic gates. These biosensors can effectively realize logic-gated biomedical functions [41]. Isothermal nucleic acid amplification provides efficient amplification without the need for sophisticated temperature cycling equipment and has extremely high amplification efficiencies, such as SDA [42], rollover amplification [43], exponential amplification [44], and loop-mediated isothermal amplification [45].

**Figure 4.**
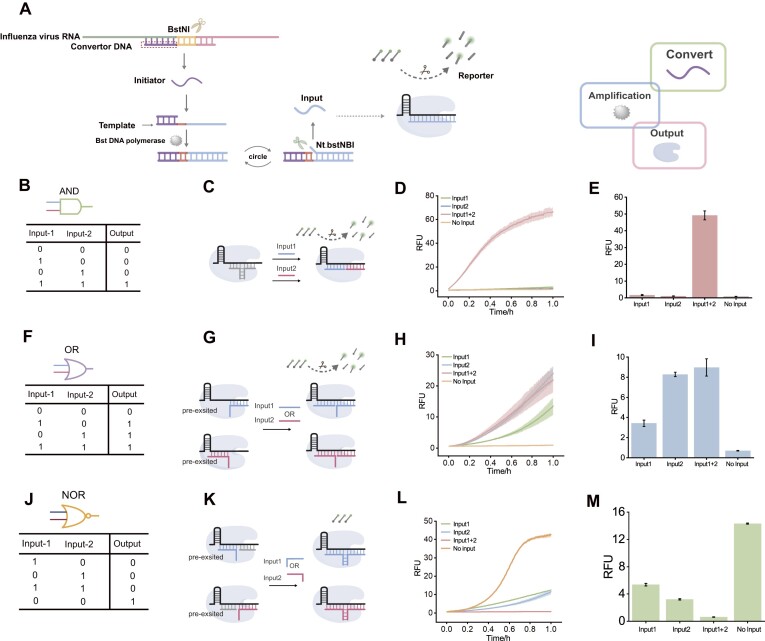
(**A**) Schemes for influenza RNA transformation, SDA amplification, and signal output. Influenza viruses A and B produce output strand 1 and 2, respectively. (**B**) Truth table of “AND” gate. (**C**) “AND” logic gate implementation. Output 1 and 2 synergistically activate Cas. (**D**) Fluorescence plot of “AND” logic gate and bar graph (**E**). (**F**) Truth table of “OR” gate. (**G**) “OR” logic gate implementation. Output 1 and 2 synergistically activate Cas with preloaded activators, respectively. (**H**) Fluorescence plot of “OR” logic gate and bar graph (**I**). (**J**) Truth table of “NOR” gate. (**K**) “NOR” logic gate implementation. The overhanging domains of the output and preloaded chains are complementary thereby activating the Cas. (**L**) Fluorescence plot of “NOR” logic gate and bar graph (**M**). The concentration of preloaded activators is 50 nM, RNP is 40 nM, and the reporter is 250 nM.

We coupled isothermal amplification with the CRISPR/Cas12a system to achieve logical identification of influenza A and B viruses. SDA was used as the converter in this system. The logical analysis process involves several steps: recognition of viral RNA, SDA amplification, and logic gate signal output (Fig. 4A). Specifically, different conversion DNA are designed to hybridize with viral RNA sequences, forming RNA/DNA heteroduplexes with BstNI endonuclease recognition sites. A short initiator is released upon the action of the restriction endonucleases. This initiator then hybridizes with the corresponding SDA amplification template and is extended by Bst DNA polymerase. The resulting dsDNA is cleaved by Nt.BstNBI to release the input. PAGE results confirm the successful generation of both the initiator and input (Supplementary Figs S18–S20).

When both influenza A and B virus RNAs are present simultaneously, they are converted to form initiator A and B, respectively, leading to the generation of input 1 and input 2 through SDA. We first constructed the “AND” gate (Fig. 4C). The truth table for the “AND” gate is shown in Fig. 4B. In the absence of input, the overhang domain binds to crRNA and prevents the activation of the *trans*-cleavage activity of Cas12a. However, when both input 1 and input 2 are present, the complex containing the overhang domain is displaced, thereby activating the *trans*-cleavage activity of Cas12a and producing fluorescence (Fig. 4D and E). Next, we constructed the “OR” gate (Fig. 4G). The truth table for the “OR” gate is presented in Fig. 4F. In this case, Cas12a cannot be activated by a single overhang domain in the absence of input. However, when either input 1 or input2 is introduced, the *trans*-cleavage activity of Cas12a is activated, resulting in fluorescence (Fig. 4H–I). Then the “NOR” gate was constructed (Fig. 4K). The truth table for the “NOR” gate is shown in Fig. 4J. In the absence of input, the activator with single overhang does not inhibit the activation of Cas12a, allowing for fluorescence production. When input1 or input2 is added, it replaces the gray activator, inhibiting the activity of Cas12a (Fig. 4L and M). Additionally, the synthetic input has successfully constructed all three logic gates, as illustrated in Supplementary Fig. S21.

### Universal CRISPR/Cas12a system for multi-enzyme assays

Human apurinic/apyrimidinic endonuclease 1 (APE1) is a multifunctional enzyme in human cells, which plays a key role in the base excision repair (BER) pathway by recognizing the apurinic/apyrimidinic (AP) site in dsDNA [46]. It has been shown that the development of various tumors is accompanied by changes in APE1 expression levels or intracellular distribution [47]. Therefore, effective monitoring of intracellular APE1 expression is beneficial for tumor diagnosis and further treatment. RNase H, as an important member of the nucleotidyl transferase superfamily, is widely involved in maintaining eukaryotic genome replication and genome stability by cleaving RNA domains in RNA/DNA hybrids [48, 49]. RNase H is important during viral replication, particularly retroviruses, and is attributed to its function of specifically hydrolyzing RNA in DNA–RNA duplexes and producing suitable primers for their DNA synthesis. UDG is an essential DNA n-glycosylase for humans that recognizes and excises U and generates a base-free site (AP site) for completing the entire BER pathway coordinated with other repair enzymes [50]. Related studies have demonstrated that dysregulation of UDG may directly interfere with normal function in maintaining genetic integrity, leading to a variety of human diseases including neurodegeneration, Bloom syndrome, human immunodeficiency, and cancer. Therefore, this strategy was used to detect three enzymes: APE1, RNase H, and UDG.

**Figure 5.**
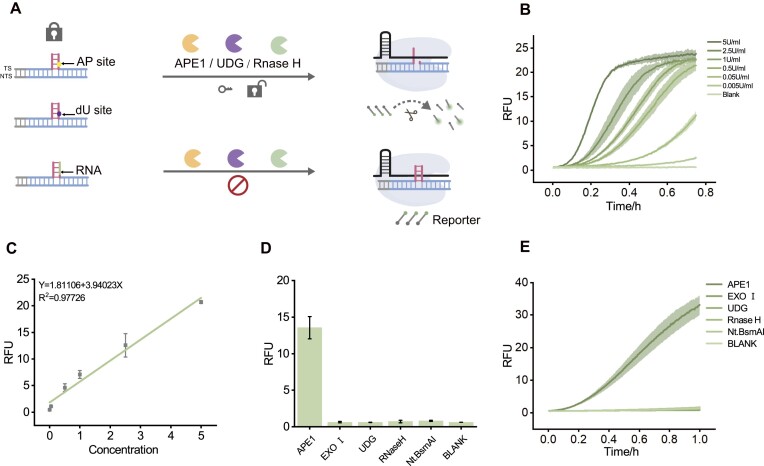
(**A**) Scheme for enzyme detection. Different enzymes cut off the corresponding overhanging domains to activate Cas. (**B**) Fluorescence plot of APE1 detection at different concentrations. (**C**) The linear plot of APE1. (**D**, **E**) Specific detection of APE1. The concentration of RNP is 40 nM and the reporter is 250 nM. The concentration of the digested complex in the final reaction system is 250 nM.

For APE1 detection, we designed the AP site into the overhang domain of the TS. Initially, the dsDNA with 8-bp overhangs cannot activate the *trans*-cleavage activity of Cas12a (Supplementary Fig. S22). Upon adding APE1, the AP site is cleaved, releasing part of the overhanging domain and restoring the *trans*-cleavage activity of Cas12a, leading to the generation of a fluorescent signal. This method enables detection of APE1 at concentrations as low as 0.005 U/ml (Fig. 5B), and show good linearity (Fig. 5C). Our system demonstrated strong selectivity for APE1 compared with other nucleases (Fig. 5D and E). Additionally, we achieved detection of the UDG enzyme by substituting the AP site with a dU site (Supplementary Fig. S23). RNase H was detected by replacing the released overhang domain with RNA (Supplementary Fig. S24). These results illustrate the flexibility of this strategy for detecting various enzyme.

## Discussion

In constructing nucleic acid-based molecular reaction networks, the immediate activation of CRISPR poses a challenge, as it can destroy the entire system. Consequently, CRISPR is often integrated as the final component, which has inherent limitations. Delayed activation is crucial to avoid premature or excessive enzyme activity, especially for applications in gene therapy and drug delivery that require slow, sustained release of Cas12a. The flexible regulation of Cas activity could directly address these challenges, leading to more effective and controlled therapeutic outcomes.

In conclusion, this method demonstrates a simple and effective strategy to control CRISPR/Cas12a to meet the more diverse and nuanced needs of the biological field. By simply adjusting the position, length, and complementarity of the overhanging domain, the *trans*-cleavage activity of CRISPR/Cas12a can be regulated, and the triggering time of the activity can be delayed while maintaining a certain activity, i.e. spontaneous delayed activation. This regulation can be achieved in single-strand activation mode and double-strand activation mode. Overall, changes in the position of the overhanging domain have the greatest impact on the rate of Cas. The overhanging domain close to PAM significantly slows down the rate of *trans*-cleavage, while the overhanging domain far away from PAM accelerates the rate of *trans*-cleavage. The middle position of the overhanging domain has less effect on the rate of Cas, making it easier to achieve delayed activation. Based on this, adjusting the length of the overhanging domain can achieve regulation of the activation time while keeping the *trans*-cleavage rate almost unchanged. Further, adjusting the length of a single overhang can realize short time regulation in minutes, and adjusting the length of double overhangs can realize long time regulation in tens of minutes. The time delay can be further enhanced when the overhanging domains are complementary.

The activator containing the overhanging domain enables precise activation timing and regulation of Cas12a and successfully achieving dynamic control of Cas12a activity, programmable release of ATP, logical operations, and universal enzyme detection. This dynamic and flexible CRISPR regulation strategy will provide new insights into the CRISPR/Cas12a system, with the potential to expand applications in various fields. For molecular detection, delayed activation of enzymes can initiate reactions only when the target analyte reaches a specific threshold concentration. This mechanism enhances signal intensity, as the enzyme is activated only in the presence of the target, avoiding interference from background noise and improving sensitivity. Moreover, enzymes can be designed to be sensitive only to specific analytes or conditions. This selectivity prevents cross-reactivity, thereby increasing the specificity of the sensor and enabling effective differentiation of multiple analytes in complex samples. In therapeutic applications, the delayed mechanism can be linked to drug delivery, where delayed drug release helps maintain therapeutic concentrations. This allows for targeted release at specific times and locations, thereby enhancing drug efficacy and safety. In bioengineering, biological systems based on delayed activation can enable cells to initiate gene expression at specific time points or under particular conditions. This facilitates more precise control of biological functions and benefits the regulation of gene expression.

## Supplementary Material

gkaf117_Supplemental_File

## Data Availability

All data supporting the findings of this study are available within the article and its supplementary information or will be made available from the authors upon request.

## References

[1] Barrangou R , Fremaux C, Deveau H et al. CRISPR provides acquired resistance against viruses in prokaryotes. Science. 2007; 315:1709–12. 10.1126/science.1138140. 17379808

[2] Brouns SJJ , Jore MM, Lundgren M et al. Small CRISPR RNAs guide antiviral defense in prokaryotes. Science. 2008; 321:960–4. 10.1126/science.1159689. 18703739 PMC5898235

[3] Makarova KS , Wolf YI, Iranzo J et al. Evolutionary classification of CRISPR–Cas systems: a burst of class 2 and derived variants. Nat Rev Micro. 2020; 18:67–83. 10.1038/s41579-019-0299-x. PMC8905525 31857715

[4] Pinilla-Redondo R , Mayo-Muñoz D, Russel J et al. Type IV CRISPR–Cas systems are highly diverse and involved in competition between plasmids. Nucleic Acids Res. 2020; 48:2000–12. 10.1093/nar/gkz1197. 31879772 PMC7038947

[5] Zetsche B , Gootenberg JS, Abudayyeh OO et al. Cpf1 is a single RNA-guided endonuclease of a class 2 CRISPR–Cas system. Cell. 2015; 163:759–71. 10.1016/j.cell.2015.09.038. 26422227 PMC4638220

[6] Swarts DC , Jinek M Mechanistic insights into the *cis*- and *trans*-acting DNase activities of Cas12a. Mol Cell. 2019; 73:589–600. 10.1016/j.molcel.2018.11.021. 30639240 PMC6858279

[7] Gier RA , Budinich KA, Evitt NH et al. High-performance CRISPR–Cas12a genome editing for combinatorial genetic screening. Nat Commun. 2020; 11:3455 10.1038/s41467-020-17209-1. 32661245 PMC7359328

[8] Broughton JP , Deng X, Yu G et al. CRISPR–Cas12-based detection of SARS-CoV-2. Nat Biotechnol. 2020; 38:870–4. 10.1038/s41587-020-0513-4. 32300245 PMC9107629

[9] Wang J , Lu A, Bei J et al. CRISPR/ddCas12a-based programmable and accurate gene regulation. Cell Discov. 2019; 5:15 10.1038/s41421-019-0085-y. 30886738 PMC6411887

[10] Chen S , Wang R, Peng S et al. PAM-less conditional DNA substrates leverage *trans*-cleavage of CRISPR–Cas12a for versatile live-cell biosensing. Chem Sci. 2022; 13:2011–20. 10.1039/D1SC05558E. 35308851 PMC8848855

[11] Ling X , Chang L, Chen H et al. Improving the efficiency of CRISPR–Cas12a-based genome editing with site-specific covalent Cas12a–crRNA conjugates. Mol Cell. 2021; 81:4747–56. 10.1016/j.molcel.2021.09.021. 34648747

[12] Wu Y , Chang D, Chang Y et al. Nucleic acid enzyme-activated CRISPR–Cas12a with circular CRISPR RNA for biosensing. Small. 2023; 19:e2303007 10.1002/smll.202303007. 37294164

[13] Hu M , Qiu Z, Bi Z et al. Photocontrolled crRNA activation enables robust CRISPR–Cas12a diagnostics. Proc Natl Acad Sci USA. 2022; 119:e2202034119 10.1073/pnas.2202034119. 35727982 PMC9245704

[14] Deng H , Xu H, Wang Y et al. G-quadruplex-based CRISPR photoswitch for spatiotemporal control of genomic modulation. Nucleic Acids Res. 2023; 51:4064–77. 10.1093/nar/gkad178. 36912089 PMC10164585

[15] Liu X , Cui S, Qi Q et al. G-quadruplex-guided RNA engineering to modulate CRISPR-based genomic regulation. Nucleic Acids Res. 2022; 50:11387–400. 10.1093/nar/gkac870. 36263801 PMC9638906

[16] Oesinghaus L , Simmel FC Switching the activity of Cas12a using guide RNA strand displacement circuits. Nat Commun. 2019; 10:2092 10.1038/s41467-019-09953-w. 31064995 PMC6504869

[17] Hu Z , Sun A, Yang J et al. Regulation of the CRISPR–Cas12a system by methylation and demethylation of guide RNA. Chem Sci. 2023; 14:5945–55. 10.1039/D3SC00629H. 37293662 PMC10246701

[18] Nguyen LT , Smith BM, Jain PK Enhancement of *trans*-cleavage activity of Cas12a with engineered crRNA enables amplified nucleic acid detection. Nat Commun. 2020; 11:4906 10.1038/s41467-020-18615-1. 32999292 PMC7528031

[19] Liu TY , Knott GJ, Smock DCJ et al. Accelerated RNA detection using tandem CRISPR nucleases. Nat Chem Biol. 2021; 17:982–8. 10.1038/s41589-021-00842-2. 34354262 PMC10184463

[20] Fei X , Lei C, Ren W et al. Regulating the *trans*-cleavage activity of CRISPR/Cas12a by using an elongation-caged single-stranded DNA activator and the biosensing applications. Anal Chem. 2023; 95:12169–76. 10.1021/acs.analchem.3c02471. 37531567

[21] Li Q , Song Z-L, Zhang Y et al. Synergistic incorporation of two ssDNA activators enhances the *trans*-cleavage of CRISPR/Cas12a. Anal Chem. 2023; 95:8879–88. 10.1021/acs.analchem.3c00414. 37252785

[22] Rananaware SR , Vesco EK, Shoemaker GM et al. Programmable RNA detection with CRISPR–Cas12a. Nat Commun. 2023; 14:5409 10.1038/s41467-023-41006-1. 37669948 PMC10480431

[23] Hu M , Cheng X, Wu T Modular CRISPR/Cas12a synergistic activation platform for detection and logic operations. Nucleic Acids Res. 2024; 52:7384–96. 10.1093/nar/gkae470. 38828769 PMC11229313

[24] Yosef N , Regev A Impulse control: temporal dynamics in gene transcription. Cell. 2011; 144:886–96. 10.1016/j.cell.2011.02.015. 21414481 PMC3148525

[25] Purvis JE , Lahav G Encoding and decoding cellular information through signaling dynamics. Cell. 2013; 152:945–56. 10.1016/j.cell.2013.02.005. 23452846 PMC3707615

[26] Li S , Jiang Q, Liu S et al. A DNA nanorobot functions as a cancer therapeutic in response to a molecular trigger *in vivo*. Nat Biotechnol. 2018; 36:258–64. 10.1038/nbt.4071. 29431737

[27] Srinivas N , Parkin J, Seelig G et al. Enzyme-free nucleic acid dynamical systems. Science. 2017; 358:eaal2052 10.1126/science.aal2052. 29242317

[28] van Roekel HWH , Meijer LHH, Masroor S et al. Automated design of programmable enzyme-driven DNA circuits. ACS Synth Biol. 2015; 4:735–45. 10.1021/sb500300d. 25365785

[29] Green LN , Subramanian HKK, Mardanlou V et al. Autonomous dynamic control of DNA nanostructure self-assembly. Nat Chem. 2019; 11:510–20. 10.1038/s41557-019-0251-8. 31011170

[30] Bucci J , Irmisch P, Del Grosso E et al. Orthogonal enzyme-driven timers for DNA strand displacement reactions. J Am Chem Soc. 2022; 144:19791–8. 10.1021/jacs.2c06599. 36257052 PMC9634797

[31] Deng J , Walther A Fuel-driven transient DNA strand displacement circuitry with self-resetting function. J Am Chem Soc. 2020; 142:21102–9. 10.1021/jacs.0c09681. 33322910 PMC7612460

[32] Wu Y , Luo W, Weng Z et al. A PAM-free CRISPR/Cas12a ultra-specific activation mode based on toehold-mediated strand displacement and branch migration. Nucleic Acids Res. 2022; 50:11727–37. 10.1093/nar/gkac886. 36318259 PMC9723625

[33] Zhao R , Luo W, Wu Y et al. Unmodificated stepless regulation of CRISPR/Cas12a multi-performance. Nucleic Acids Res. 2023; 51:10795–807. 10.1093/nar/gkad748. 37757856 PMC10602922

[34] Seeman NC , Sleiman HF DNA nanotechnology. Nat Rev Mater. 2017; 3:17068 10.1038/natrevmats.2017.68.

[35] Nickels PC , Wünsch B, Holzmeister P et al. Molecular force spectroscopy with a DNA origami-based nanoscopic force clamp. Science. 2016; 354:305–7. 10.1126/science.aah5974. 27846560 PMC6546592

[36] Hu Y , Cecconello A, Idili A et al. Triplex DNA nanostructures: from basic properties to applications. Angew Chem Int Ed. 2017; 56:15210–33. 10.1002/anie.201701868. 28444822

[37] Del Grosso E , Irmisch P, Gentile S et al. Dissipative control over the Toehold-mediated DNA strand displacement reaction. Angew Chem Int Ed. 2022; 61:e202201929 10.1002/anie.202201929. PMC9324813 35315568

[38] Del Grosso E , Franco E, Prins LJ et al. Dissipative DNA nanotechnology. Nat Chem. 2022; 14:600–13. 10.1038/s41557-022-00957-6. 35668213

[39] Zhong H , Li X, Yu N et al. Fine-tuning the sequential drug release of nano-formulated mutual prodrugs dictates the combination effects. Chem Sci. 2023; 14:3789–99. 10.1039/D3SC00550J. 37035705 PMC10074403

[40] Awada HK , Johnson NR, Wang Y Sequential delivery of angiogenic growth factors improves revascularization and heart function after myocardial infarction. J Control Release. 2015; 207:7–17. 10.1016/j.jconrel.2015.03.034. 25836592 PMC4430430

[41] Yin F , Wang F, Fan C et al. Biosensors based on DNA logic gates. VIEW. 2021; 2:20200038 10.1002/VIW.20200038.

[42] Walker GT , Fraiser MS, Schram JL et al. Strand displacement amplification–an isothermal, *in vitro* DNA amplification technique. Nucleic Acids Res. 1992; 20:1691–6. 10.1093/nar/20.7.1691. 1579461 PMC312258

[43] Kang X , Wu C, Alibakhshi MA et al. Nanopore-based fingerprint immunoassay based on rolling circle amplification and DNA fragmentation. ACS Nano. 2023; 17:5412–20. 10.1021/acsnano.2c09889. 36877993 PMC10629239

[44] Carter JG , Orueta Iturbe L, Duprey J-LHA et al. Ultrarapid detection of SARS-CoV-2 RNA using a reverse transcription-free exponential amplification reaction, RTF-EXPAR. Proc Natl Acad Sci USA. 2021; 118:e2100347118 10.1073/pnas.2100347118. 34400545 PMC8536344

[45] Soroka M , Wasowicz B, Rymaszewska A Loop-mediated isothermal amplification (LAMP): the better sibling of PCR?. Cells. 2021; 10:1931 10.3390/cells10081931. 34440699 PMC8393631

[46] Liu T-C , Lin C-T, Chang K-C et al. APE1 distinguishes DNA substrates in exonucleolytic cleavage by induced space-filling. Nat Commun. 2021; 12:601 10.1038/s41467-020-20853-2. 33504804 PMC7841161

[47] Xiang Z , Zhao J, Qu J et al. A multivariate-gated DNA nanodevice for spatioselective imaging of pro-metastatic targets in extracellular microenvironment. Angew Chem Int Ed. 2022; 61:e202111836 10.1002/anie.202111836. 34779093

[48] Camino LP , Dutta A, Barroso S et al. DICER ribonuclease removes harmful R-loops. Mol Cell. 2023; 83:3707–19. 10.1016/j.molcel.2023.09.021. 37827159 PMC11034902

[49] Lee H , Cho H, Kim J et al. RNase H is an exo- and endoribonuclease with asymmetric directionality, depending on the binding mode to the structural variants of RNA:DNA hybrids. Nucleic Acids Res. 2022; 50:1801–14. 10.1093/nar/gkab1064. 34788459 PMC8886854

[50] Shiraishi M , Ishino S, Heffernan M et al. The mesophilic archaeon methanosarcina acetivorans counteracts uracil in DNA with multiple enzymes: endoQ, ExoIII, and UDG. Sci Rep. 2018; 8:15791 10.1038/s41598-018-34000-x. 30361558 PMC6202378

