# Supplementary material for: Regulation of CRISPR trans-cleavage activity by an overhanging activator
Source: Nucleic Acids Res. 2025 Feb 24;53(4):gkaf117. doi: 10.1093/nar/gkaf117 (PMC11850226; doi:10.1093/nar/gkaf117)
Supplement: gkaf117_Supplemental_File [file gkaf117_supplemental_file.pdf]

Supporting information

Regulation of Cas12a trans-cleavage activity by an overhanging activator

Na Yin[1, †], Hongyan Yu[1, †], Li Zhang[1, †], Fei Luo[1], Weitao Wang[1], Xiaole Han[1], Yu He[1], Yiqi Zhang[1], You Wu[1], Jiu Pu[1], Tong Feng[1], Gang Yang[3, *], Tingmei Chen[1, *], Guoming Xie[1,2 *]
[1]Key Laboratory of Clinical Laboratory Diagnostics (Chinese Ministry of Education), College of Laboratory Medicine, Chongqing Medical University, Chongqing, 400016, P.R China.
[2] Western Institute of Digital-Intelligent Medicine，Chongqing 401329, China.
[3]Department of Neurosurgery, The First Affiliated Hospital of Chongqing Medical University, Chongqing, 400016, P.R China.

*To whom correspondence should be addressed. Tel: +86 23 68485240; Email: guomingxie@cqmu.edu.cn
Correspondence may also be addressed to Tingmei Chen. Email: tingmeichen@cqmu.edu.cn
Correspondence may also be addressed to Gang Yang. Email: gangyang@hospital.cqmu.edu.cn
†The first three authors should be regarded as Joint First Authors.

Table S1 The sequences of the DNA used in the study

Strand Name	Sequence (5′→3′)
The effect of activators with overhanging domains on trans-cleavage activity in single-stranded activation mode	
α-crRNA	UAAUUUCUACUAAGUGUAGAUUAAGUUCAAGGUGUG CGCAAUG
α-crRNA-T	CATTGCGCACACCTTGAACTTA
α-crRNA sL-in	TTTTTTTTTTCCTTGAACTTA
α-crRNA sR-in	ATTGCGCACATTTTTTTTTT
β-crRNA	UAAUUUCUACUAAGUGUAGAUAGUAGAACCAUG UGUGCACCUGG
β-crRNA-T	CCAGGTGCACACATGGTTCTACT
β-crRNA sL-in	TTTTTTTTTTCATGGTTCTACT
β-crRNA sR-in	CCAGGTGCACATTTTTTTTTT
γ-crRNA	UAAUUUCUACUAAGUGUAGAUCGAUGUGGAGU GUGAGCAUGCA
γ-crRNA-T	TGCATGCTCACACTCCACATCG
γ-crRNA sL-in	TTTTTTTTTTACTCCACATCG
γ-crRNA sR-in	TGCATGCTCACTTTTTTTTTT
sL-7	GAACTTA
sL-8	TGAACTTA
sL-9	TTGAACTTA
sL-10	CTTGAACTTA
sL-11	CCTTGAACTTA
sL-12	ACCTTGAACTTA
sL-13	CACCTTGAACTTA
sL-14	ACACCTTGAACTTA
sL-15	CACACCTTGAACTTA
sR-7	CATTGCG
sR-8	CATTGCGC
sR-9	CATTGCGCA
sR-10	CATTGCGCAC
sR-11	CATTGCGCACA
sR-12	CATTGCGCACAC
sR-13	CATTGCGCACACC
sR-14	CATTGCGCACACCT
sR-15	CATTGCGCACACCTT
sL-o-7	GAACTTATTTTTTTTTT
sL-o-8	TGAACTTATTTTTTTTTT
sL-o-9	TGAACTTATTTTTTTTTT
sL-o-10	CTTGAACTTATTTTTTTTTT
sL-o-11	CCTTGAACTTATTTTTTTTTT

sL-o-12	ACCTTGAACTTATTTTTTTTTT
sL-o-13	CACCTTGAACTTA TTTTTTTTTT
sL-o-14	ACACCTTGAACTTA TTTTTTTTTT
sL-o-15	CACACCTTGAACTTA TTTTTTTTTT
sR-o-7	TTTTTTTTTTCATTGCG
sR-o-8	TTTTTTTTTTCATTGCGC
sR-o-9	TTTTTTTTTTCATTGCGCA
sR-o-10	TTTTTTTTTTCATTGCGCAC
sR-o-11	TTTTTTTTTTCATTGCGCACA
sR-o-12	TTTTTTTTTTCATTGCGCACAC
sR-o-13	TTTTTTTTTTCATTGCGCACACC
sR-o-14	TTTTTTTTTTCATTGCGCACACCT
sR-o-15	TTTTTTTTTTCATTGCGCACACCTT
sL-in-7	TTTTTTTTTTGAACTTA
sL-in-8	TTTTTTTTTTTGAACTTA
sL-in-9	TTTTTTTTTTTTGAACTTA
sL-in-10	TTTTTTTTTTCTTGAACTTA
sL-in-11	TTTTTTTTTTCCTTGAACTTA
sL-in-12	TTTTTTTTTTACCTTGAACTTA
sL-in-13	TTTTTTTTTTCACCTTGAACTTA
sL-in-14	TTTTTTTTTTACACCTTGAACTTA
sL-in-15	TTTTTTTTTTCACACCTTGAACTTA
sR-in-7	CATTGCGTTTTTTTTTT
sR-in-8	CATTGCGCTTTTTTTTTT
sR-in-9	CATTGCGCATTTTTTTTTT
sR-in-10	CATTGCGCACTTTTTTTTTT
sR-in-11	ATTGCGCACATTTTTTTTTT
sR-in-12	CATTGCGCACACTTTTTTTTTT
sR-in-13	CATTGCGCACACCTTTTTTTTTT
sR-in-14	CATTGCGCACACCTTTTTTTTTTT
sR-in-15	CATTGCGCACACCTTTTTTTTTTTT
sL-in complementary 6bp	ACACACCCTTGAACTTA
sR-in complementary 6bp	CATTGCGCACAGTGTGT
sL-in complementary 8bp	ACACACACCCTTGAACTTA
sR-in complementary 8bp	CATTGCGCACAGTGTGTGT
sL-in-4nt	TTTTCCTTGAACTTA
sL-in-6nt	TTTTTTCCTTGAACTTA
sL-in-8nt	TTTTTTTTCCTTGAACTTA
sL-in-10nt	TTTTTTTTTTCCTTGAACTTA
sL-in-12nt	TTTTTTTTTTTTCCTTGAACTTA
sL-in-15nt	TTTTTTTTTTTTTTTCCTTGAACTTA
sL-in-30nt	TTTTTTTTTTTTTTTTTTTTTTTTTTTTTTCCTTGAACTTA

sR-in-4nt	CATTGCGCACATTTT
sR-in-6nt	CATTGCGCACATTTTTT
sR-in-8nt	CATTGCGCACATTTTTTTT
sR-in-10nt	CATTGCGCACATTTTTTTTTT
sR-in-15nt	CATTGCGCACATTTTTTTTTTTTTTT
sR-in-30nt	CATTGCGCACATTTTTTTTTTTTTTTTTTTTTTTTTTTTTT
Cas-reportor	HEX-TTATT-BHQ1

The effect of activators with overhanging domains on trans-cleavage activity in double-stranded activation mode

TS1	CATTGCGCACACCTTGAACTTACAAA
NTS1	TTTGTAAGTTCAAGGTGTGCGCAATG
TS	CATTGCGCACACCTTGAACTTATAAATG
NTS	CATTTATAAGTTCAAGGTGTGCGCAATG
NTS1-L-8	TTTGTAAGTTCA
NTS1-L-9	TTTGTAAGTTCAA
NTS1-L-10	TTTGTAAGTTCAAG
NTS1-L-11	TTTGTAAGTTCAAGG
NTS1-L-12	TTTGTAAGTTCAAGGT
NTS1-L-13	TTTGTAAGTTCAAGGTG
NTS1-L-14	TTTGTAAGTTCAAGGTGT
NTS-R-8	GCGCAATG
NTS-R-9	TGCGCAATG
NTS-R-10	GTGCGCAATG
NTS-R-11	TGTGCGCAATG
NTS-R-12	GTGTGCGCAATG
NTS-R-13	GGTGTGCGCAATG
NTS-R-14	AGGTGTGCGCAATG
NTS-L-8	CATTTATAAGTTCA
NTS-L-9	CATTTATAAGTTCAA
NTS-L-10	CATTTATAAGTTCAAG
NTS-L-11	CATTTATAAGTTCAAGG
NTS-L-12	CATTTATAAGTTCAAGGT
NTS-L-13	CATTTATAAGTTCAAGGTG
NTS-L-14	CATTTATAAGTTCAAGGTGT
TS1-L8	TGAACTTACAAA
TS1-L9	TTGAACTTACAAA
TS1-L10	CTTGAACTTACAAA
TS1-L11	CCTTGAACTTACAAA
TS1-L12	ACCTTGAACTTACAAA
TS1-L13	CACCTTGAACTTACAAA

TS1-L4	ACACCTTGAACTTACAAA
TS-R8	CATTGCGC
TS-R9	CATTGCGCA
TS-R10	CATTGCGCAC
TS-R11	CATTGCGCACA
TS-R12	CATTGCGCACAC
TS-R13	CATTGCGCACACC
TS-R14	CATTGCGCACACCT
NTS-L-in-8	CATTTATAAGTTCA TTTTTTTTTT
NTS-L-in-9	CATTTATAAGTTCAATTTTTTTTTT
NTS-L-in-10	CATTTATAAGTTCAAG TTTTTTTTTT
NTS-L-in-11	CATTTATAAGTTCAAGGTTTTTTTTTT
NTS-L-in-12	CATTTATAAGTTCAAGGTTTTTTTTTTT
NTS-L-in-13	CATTTATAAGTTCAAGGTGTTTTTTTTTT
NTS-L-in-14	CATTTATAAGTTCAAGGTGTTTTTTTTTTT
NTS-R-in-8	TTTTTTTTTTGCGCAATG
NTS-R-in-9	TTTTTTTTTTTGCGCAATG
NTS-R-in-10	TTTTTTTTTTGTGCGCAATG
NTS-R-in-11	TTTTTTTTTTTGTGCGCAATG
NTS-R-in-12	TTTTTTTTTTGTGTGCGCAATG
NTS-R-in-13	TTTTTTTTTTGGTGTGCGCAATG
NTS-R-in-14	TTTTTTTTTTAGGTGTGCGCAATG
NTS-L-in-4nt	CATTTATAAGTTCAAGGTTTT
NTS-L-in-6nt	CATTTATAAGTTCAAGGTTTTTT
NTS-L-in-8nt	CATTTATAAGTTCAAGGTTTTTTTT
NTS-L-in-10nt	CATTTATAAGTTCAAGGTTTTTTTTTT
NTS-L-in-15nt	CATTTATAAGTTCAAGGTTTTTTTTTTTTTTT
NTS-R-in-4nt	TTTTTGTGCGCAATG
NTS-R-in-6nt	TTTTTTTGTGCGCAATG
NTS-R-in-8nt	TTTTTTTTTGTGCGCAATG
NTS-R-in-10nt	TTTTTTTTTTTGTGCGCAATG
NTS-R-in-15nt	TTTTTTTTTTTTTTTTGTGCGCAATG
NTS-L-in complementary 6bp	CATTTATAAGTTCAAGGACACAC
NTS-L-in complementary 8bp	CATTTATAAGTTCAAGGACACACAC
NTS-L-in complementary 10bp	CATTTATAAGTTCAAGGACACACACAC
NTS-L-in complementary 15bp	CATTTATAAGTTCAAGGACACACACACACACA
NTS-R-in complementary 6bp	GTGTGTTGTGCGCAATG
NTS-R-in complementary 8bp	GTGTGTGTTGTGCGCAATG

NTS-R-in complementary 10bp	GTGTGTGTGTTGTGCGCAATG
NTS-R-in complementary 15bp	TGTGTGTGTGTGTGTTGTGCGCAATG
TS-L-in-8	TTTTTTTTTGAACTTATAAATG
TS-L-in-9	TTTTTTTTTTGAACTTATAAATG
TS-L-in-10	TTTTTTTTCTTGAACTTATAAATG
TS-L-in-11	TTTTTTTTCCTTGAACTTATAAATG
TS-L-in-12	TTTTTTTTACCTTGAACTTATAAATG
TS-L-in-13	TTTTTTTTCACCTTGAACTTATAAATG
TS-L-in-14	TTTTTTTTACACCTTGAACTTATAAATG
TS-R-in-8	CATTGCGCTTTTTTTT
TS-R-in-9	CATTGCGCATTTTTTTT
TS-R-in-10	CATTGCGCACTTTTTTTT
TS-R-in-11	CATTGCGCACATTTTTTTT
TS-R-in-12	CATTGCGCACACTTTTTTTT
TS-R-in-13	CATTGCGCACACCTTTTTTTT
TS-R-in-14	CATTGCGCACACCTTTTTTTTT
TS-L-in-2nt	TTCCTTGAACTTATAAATG
TS-L-in-6nt	TTTTTTCCTTGAACTTATAAATG
TS-L-in-8nt	TTTTTTTTCCTTGAACTTATAAATG
TS-R-in-2nt	CATTGCGCACATT
TS-R-in-6nt	CATTGCGCACATTTTTT
TS-R-in-8nt	CATTGCGCACATTTTTTTT
TS-L-in complementary 2bp	AGCCTTGAACTTATAAATG
TS-L-in complementary 6bp	AGAGAGCCTTGAACTTATAAATG
TS-L-in complementary 8bp	AGAGAGAGCCTTGAACTTATAAATG
TS-R-in complementary 2bp	CATTGCGCACACT
TS-R-in complementary 6bp	CATTGCGCACACTCTCT
TS-R-in complementary 8bp	CATTGCGCACACTCTCTCT

Programmable release of ATP and dynamic regulation

ATP-aptamer	FAM-ACCTGGGGGAGTATTTATTTATTTGCGGAGGAAGGA-BHQ1
Activator	GCTAGCTACATTGCGCACACCTTGAACTTA
Inhibitor-L	TAAGTTCAAGGACACACACAC
Inhibitor-R	GTGTGTGTGTTGTGCGCAATGTAGCTAGC

Logic gate construction and influenza virus analysis

AND-inhibitor-L	ACACACACCCTTG
AND-inhibitor-R	GCACAGTGTGTGT

OR-pre-exsited-L	TTTTTTCCTTGAACTTA
OR-pre-exsited-R	CATTGCGCACATTTTTT
NOR-pre-exsited-L1	ACACACACACACCCTTGAACTTA
NOR-pre-exsited-R1	CATTGCGCACA
NOR-pre-exsited-L2	CCTTGAACTTA
NOR-pre-exsited-R2	CATTGCGCACAACACACACACAC
AND/OR Input1	CCTTGAACTTA
AND/OR Input2	CATTGCGCACA
NOR Input1	CATTGCGCACAGTGTGTGTGTGT
NOR Input2	GTGTGTGTGTGTCCTTGAACTTA
FluA RNA	AAUGGAACAUGCUAUCCAGGAAAAUUUGCUGAUUAUGAGG
FluB RNA	AUACAGAGACGGCACCAGGAGGACCCTACAAGGUGGGGAC
Convertor-FluA	ATCAGCAAATTTTCCTGGATAGCATGTT
Convertor-FluB	CTTGTAGGGTCCTCCTGGTGCCGTCTCT
InitiatorA	ATCAGCAAATTTTCC
InitiatorB	CTTGTAGGGTCCTCC
residueA	TGGATAGCATGTT
residueB	TGGTGCCGTCTCT
AND/OR Template A	TGTGCGCAATGATATGACTCGGAAAATTTGCTGAT
NOR Template A	ACACACACACACTGTGCGCAATGATATGACTCGGAAAATTTGCTGAT
AND/OR Template B	TAAGTTCAAGGATATGACTCGGAGGACCCTACAAG
NOR Template B	TAAGTTCAAGGACACACACACACATATGACTCGGAGGACCCTACAAG

The recognition sites of restriction endonucleases：

BstNI

5′...CC▼WGG...3′
3′...GG▼WCC...5′

Nt.BstNBI
5′...GAGTCNNNN▼N...3′
3′...CTCAGNNNNN...5′

Universal CRISPR/Cas12a system for multi-enzyme assays

APE-TS-L	CGACGTAGCCTTGAACTTACAAA
APE-TS-R	CATTGCGCACAC/idSp/ACGTCG
NTS	TTTGTAAGTTCAAGGTGTGCGCAATG

UDG-TS-L	CGACGTAG CCTTGAACTTA CAAA
UDG-TS-R	CATTGCGCACA C/ ideoxyU/ACGTCG
Rnase H-TS-L	CGACGTAGCCTTGAACTTA CAAA
Rnase H-TS-R	CATTGCGCACACr（UACGUCG）

References of influenza RNA sequences：

1. Zhu,Y., Gu,X., Tang,Q., Jiang,W., Xia,R., Zhang,J., Ji,H., Qin,Y. and Wu,L. (2024)HCR-Assisted RTF-EXPAR-Based Lateral Flow Analysis for Sensitive Detection of H1N1 Influenza Virus. *Anal. Chem.*, **96**, 14116–14124.

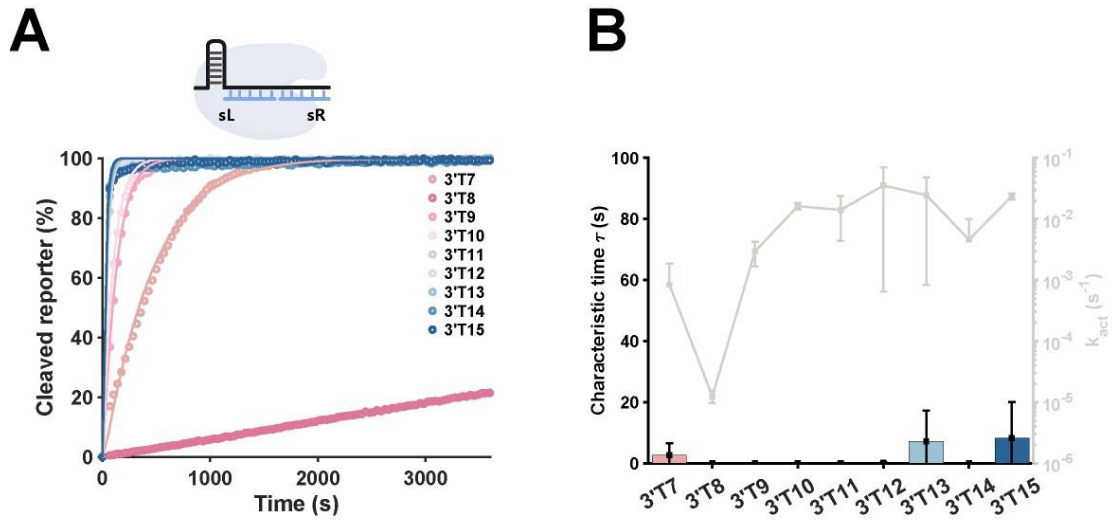

Figure S1 (A) The fluorescence kinetics of split DNA activators at different positions. The ssDNA was programmed by changing the ssDNA 3'Tx (3'represents the same untruncated 3'end as the complete ssDNA, x is the nucleotide count, and the range of x is the cleavage position of 7-15). The resulting splits are sL and sR. (B) Chracteristic time and the rate of activation.

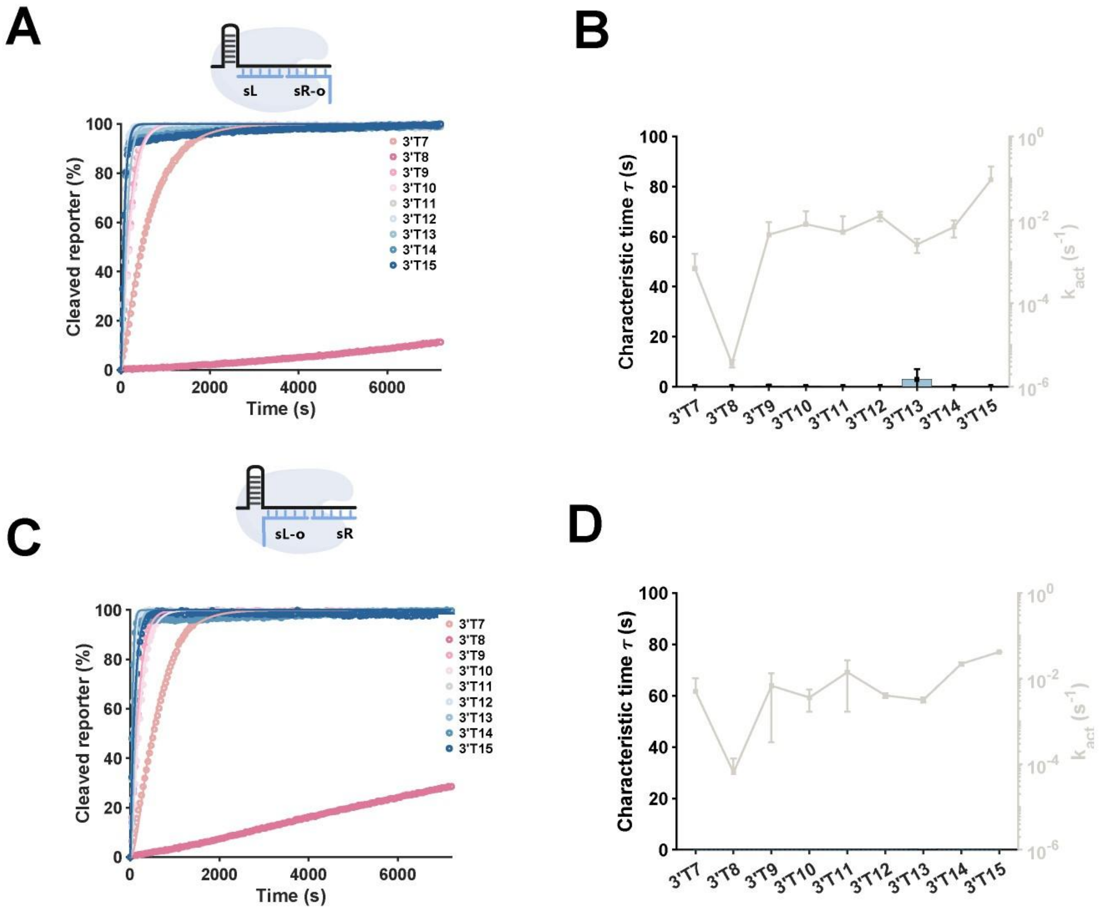

Figure S2 (A) The fluorescence kinetics of the single overhanging domain is located on the outside of sR and (B) its chracteristic time and the rate of activation. (C) The fluorescence kinetics of the single overhanging domain is located on the outside of sL and (D) its chracteristic time and the rate of activation.

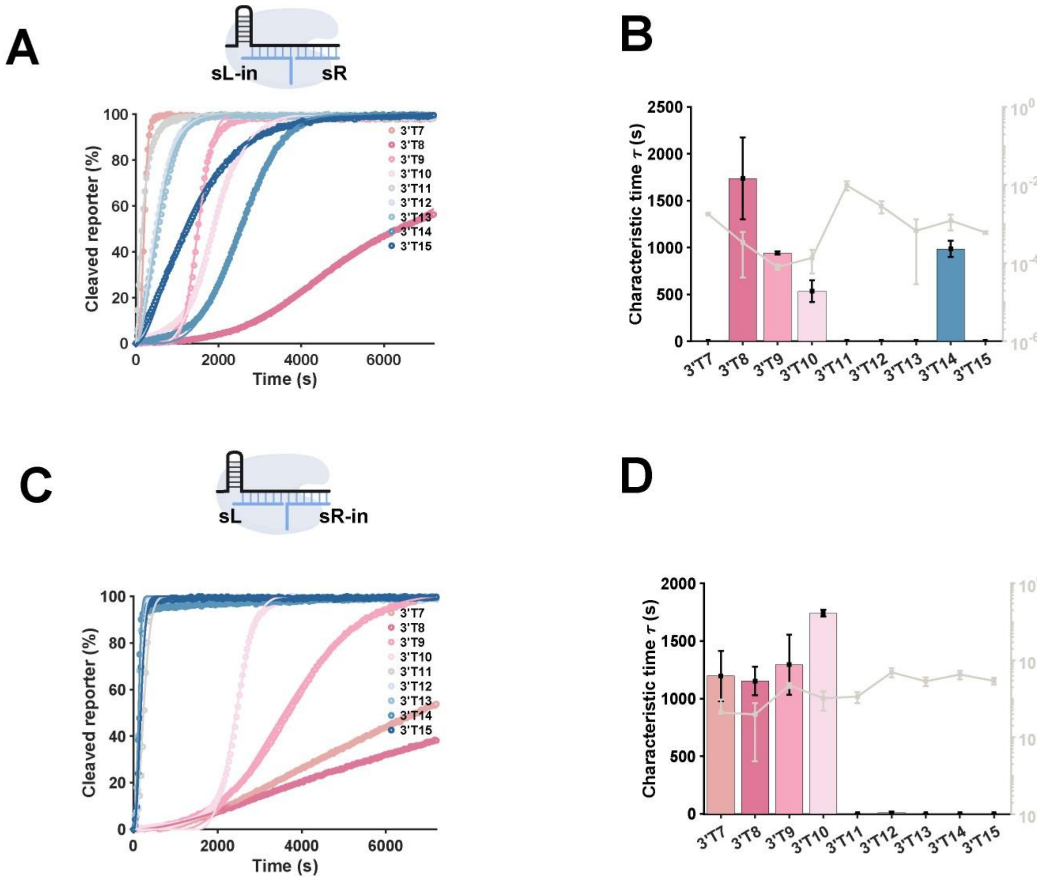

Figure S3 (A) The fluorescence kinetics of the single overhanging domain is located on the inside of sL and (B) its chracteristic time and the rate of activation. (C) The fluorescence kinetics of the single overhanging domain is located on the inside of sR and (D) its chracteristic time and the rate of activation.

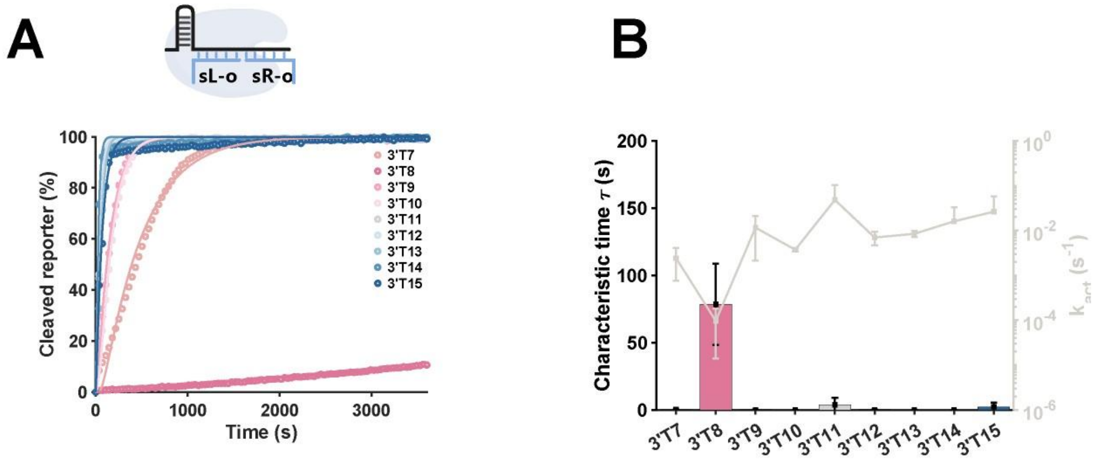

Figure S4 (A) The fluorescence kinetics of the single overhanging domain is located on the outside of sL and sR. (B) Its chracteristic time and the rate of activation.

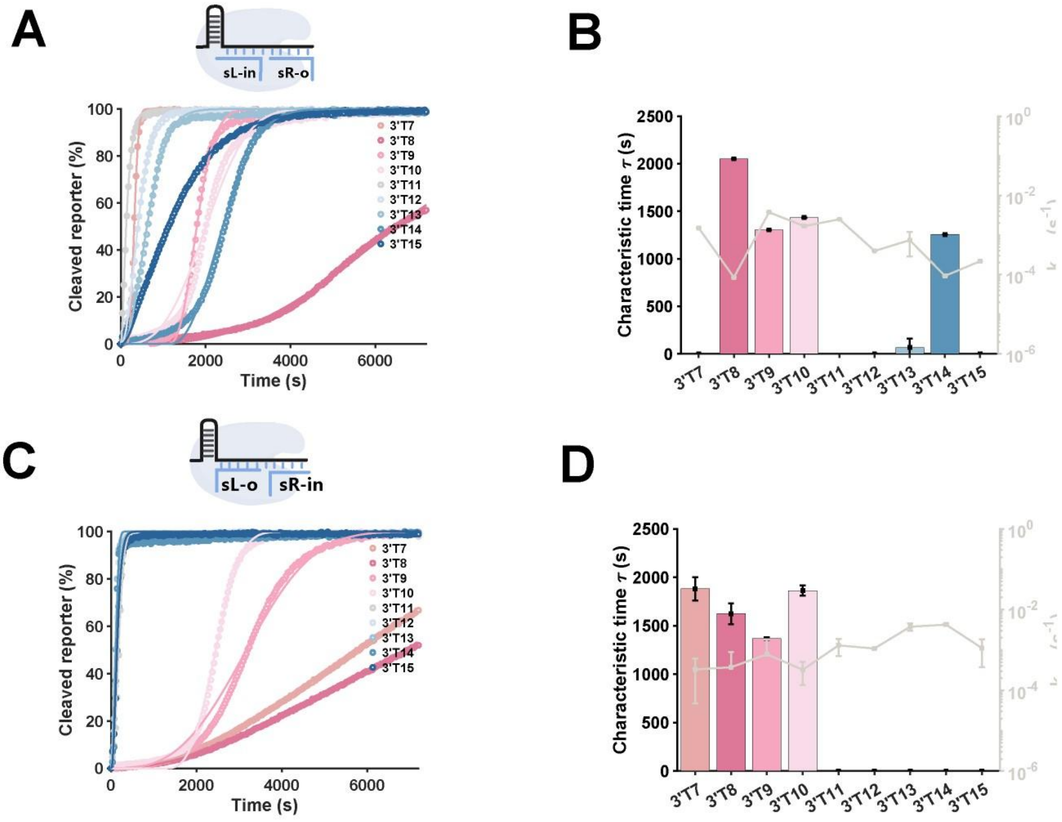

Figure S5 (A) The fluorescence kinetics of the single overhanging domain is located on the inside of sL and outside of sR. (B) Its chracteristic time and the rate of activation. (C) The fluorescence kinetics of the single overhanging domain is located on the inside of sR and outside of sL. (D) Its chracteristic time and the rate of activation.

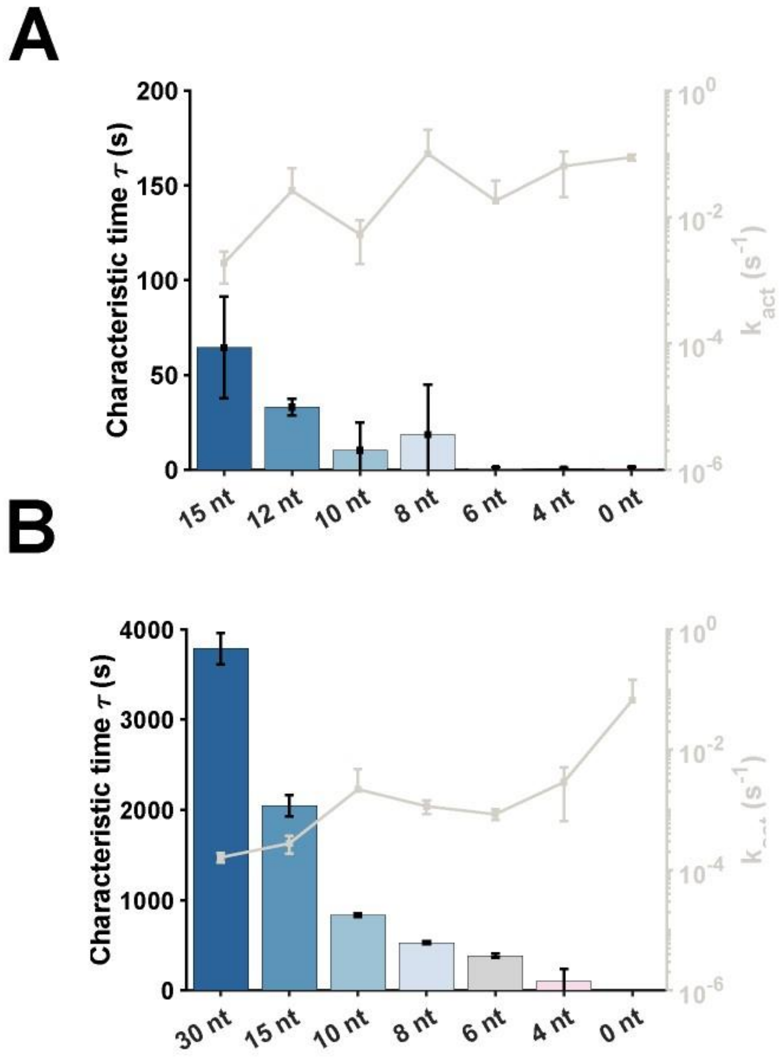

Figure S6 Chracteristic time and the rate of activation of single overhang (A) and double overhangs (B) when length changed.

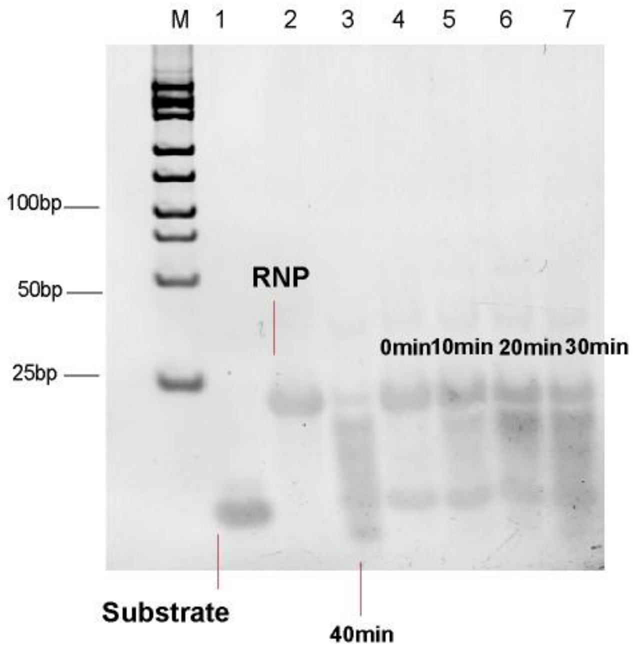

Figure S7 Page diagram of cutting products at different time periods. The double inner overhanging domains with the length of 15 nt was selected as a reference, which activated the trans-cutting activity of Cas12a at 40 min. The same system was reacted for 0min, 10min, 20min, 30min, 40min and inactivated at 80◦C, and the product was subjected to electrophoresis. Lane 1 : random cleavage of substrates. Lane 2 : RNP and double overhanging activator. Lane 3 : reaction 40min bands. Lane 4 : reaction 0min bands. Lane 5 : reaction 10min bands. Lane 6 : reaction 20 min bands. Lane 7 : reaction 30 min strips. It shows that the substrate will be cleaved after reaching activation time.

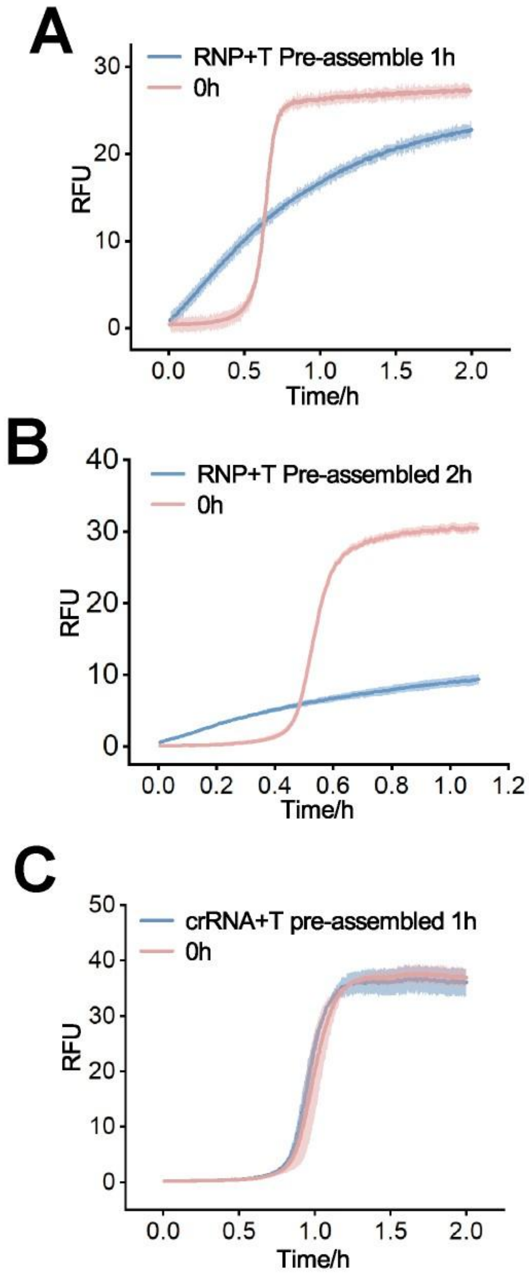

Figure S8 Fluorescence plot of RNP and activator after incubation for 1 h (A). Fluorescence plot of the reaction after incubation of RNP and activator for 2h (B). Fluorescence plot of the reaction after incubation of crRNA and activator for 2h (C).

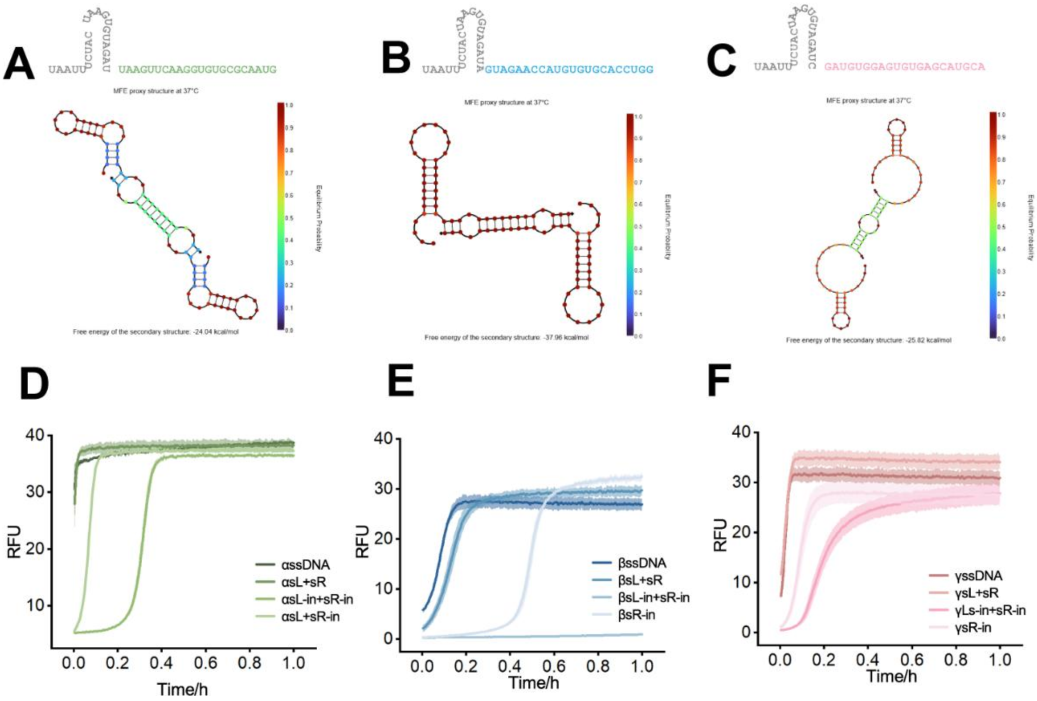

Figure S9 (A) The secondary structure of αcrRNA. (B) The secondary structure of βcrRNA. (C) The secondary structure of γcrRNA . (D-F) The fluorescence kinetics of α、β、γcrRNA activator with overhanging structure. The selected fracture position is 3'T11 or 3'T12, and the length is 10 nt.

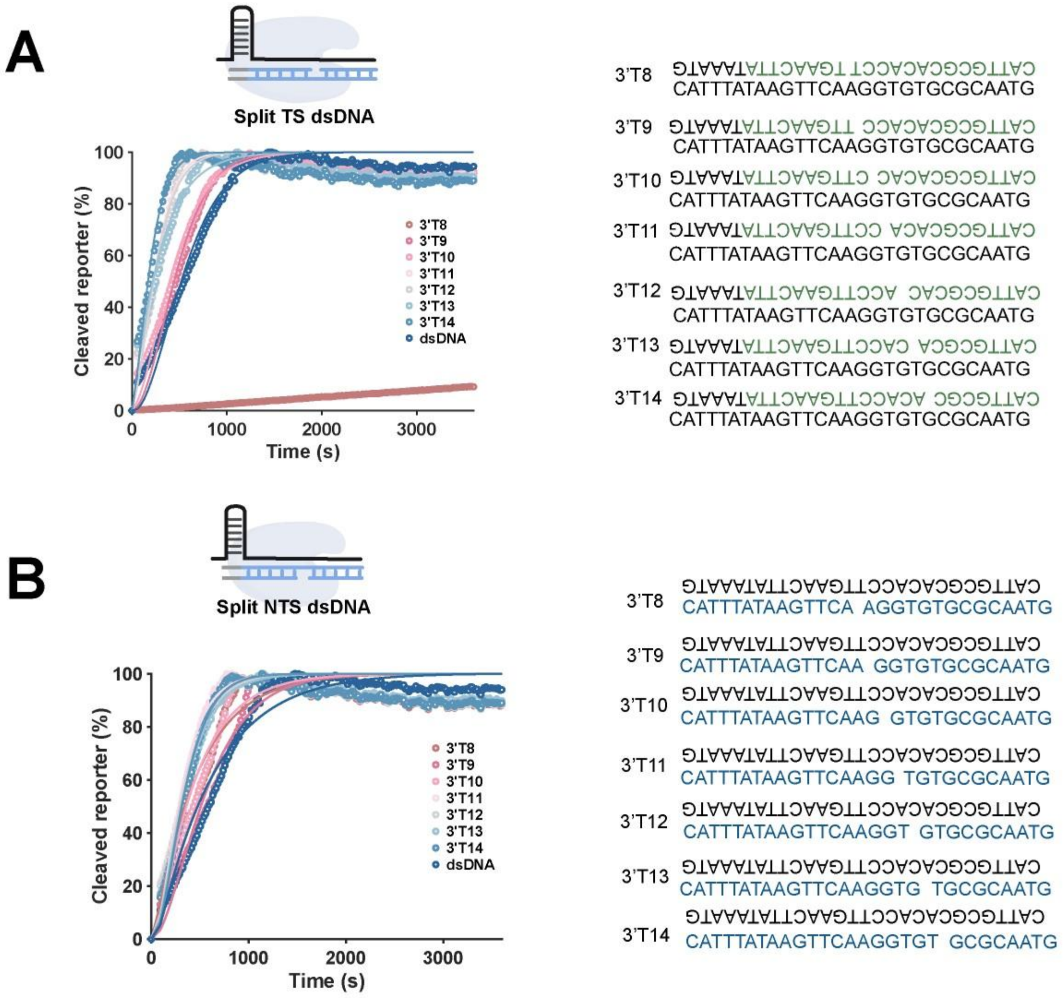

Figure S10 The fluorescence kinetics of different split modes. PAM including extra bases at the 3' end.

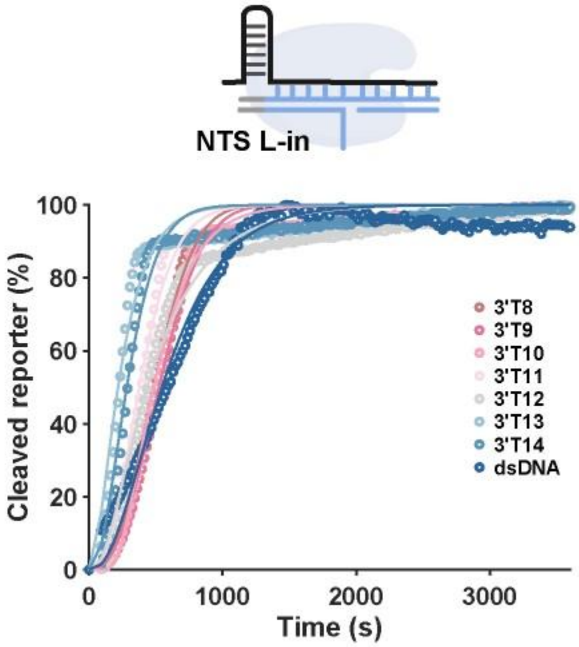

Figure S11 The fluorescence kinetics when there is an overhang inside the NTS.

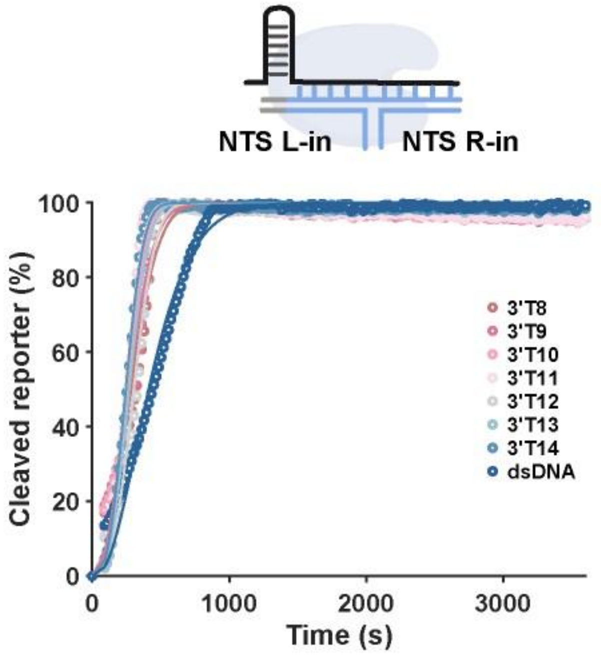

Figure S12 The fluorescence kinetics when two overhangs inside the NTS.

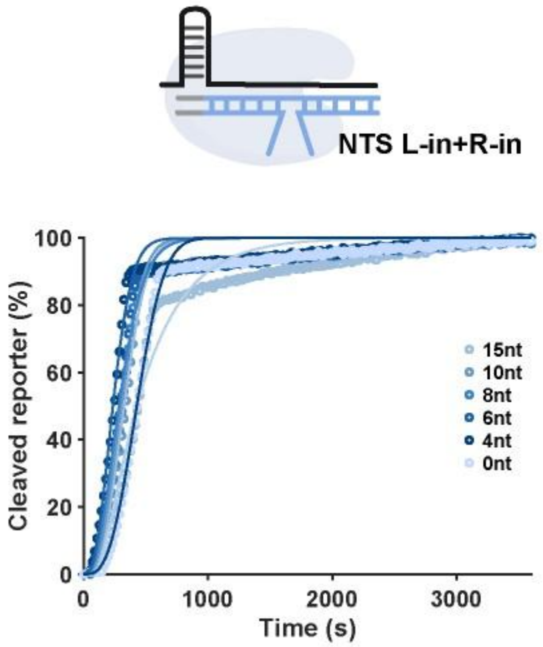

Figure S13 The fluorescence kinetics of two overhanging domains on the NTS when length is changed.

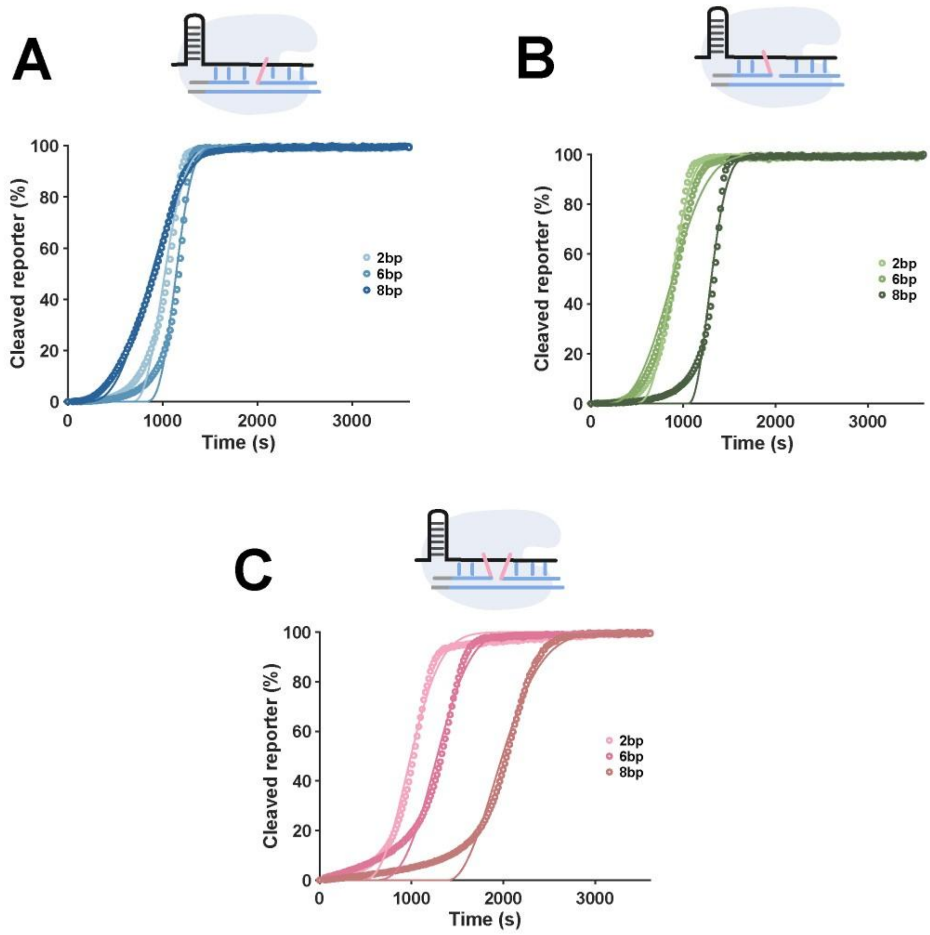

Figure S14 (A-B) The fluorescence kinetics of TS has an overhanging domain when length is changed. (C) The fluorescence kinetics of TS has two overhanging domains when length is changed.

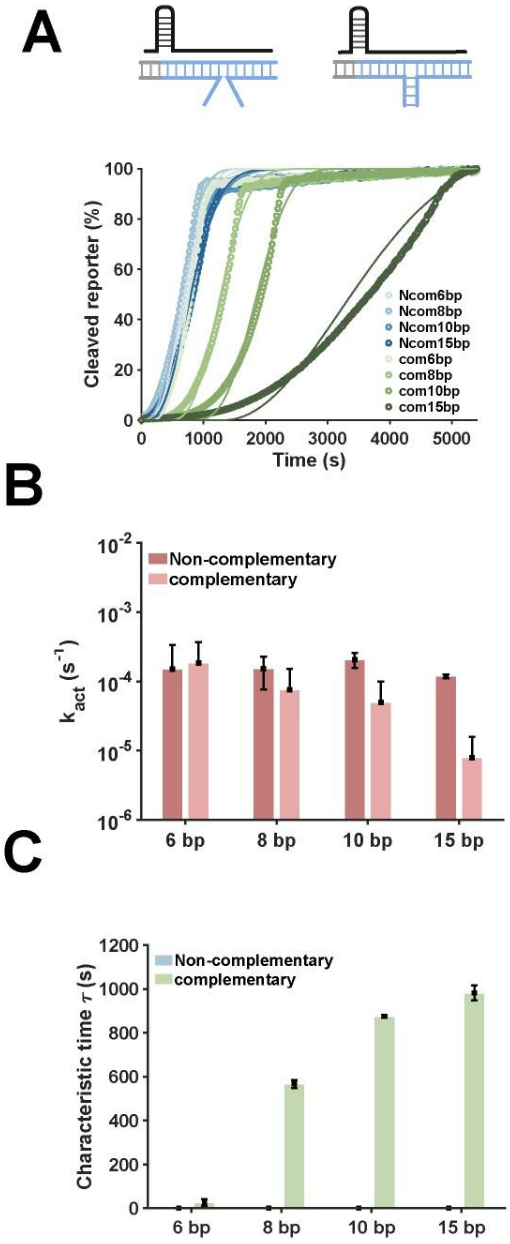

Figure S15 (A) The fluorescence kinetics of the complementary and non-complementary overhanging domains on NTS. (B) Chracteristic time. (C) Rate of activation.

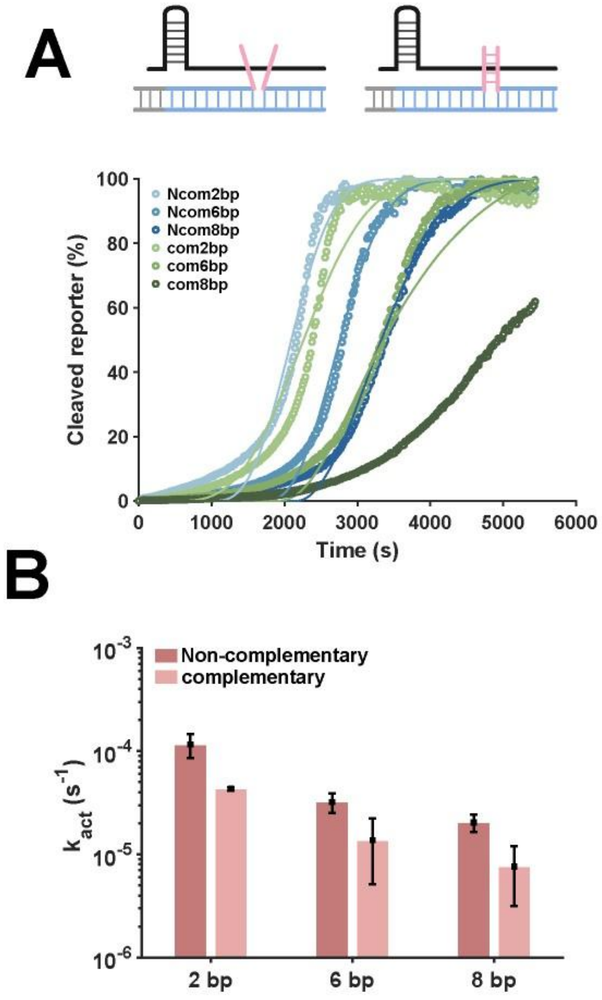

Figure S16 (A) The fluorescence kinetics of the complementary and non-complementary overhanging domains on TS. (B) Rate of activation.

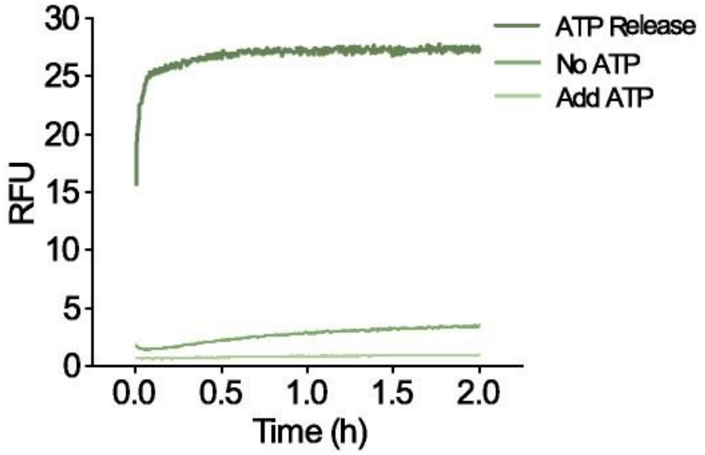

Figure S17 Fluorescence plot of the same reaction system without ATP addition, with ATP addition, and with ATP release were measured in a single tube.

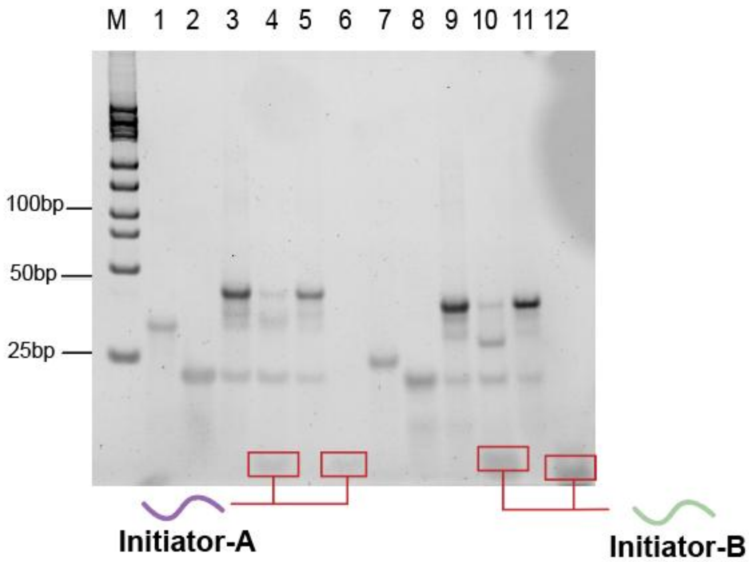

Figure S18 The PAGE verification of the transformation of influenza A RNA and influenza B RNA. Lane 1 : InfluenzaA RNA. Lane 2 : Convertor A. Lane 3 : InfluenzaA RNA + Convertor A. Lane 4 : Product inititor-A. Lane 5 : Undigested product. Lane 6 : Inititor-A control. Lane 7 : InfluenzaB RNA. Lane 8 : Convertor B. Lane 9 : InfluenzaB RNA + Convertor B. Lane 10 : Product inititor-B. Lane 11 : Undigested product. Lane 12 : Inititor-B control.

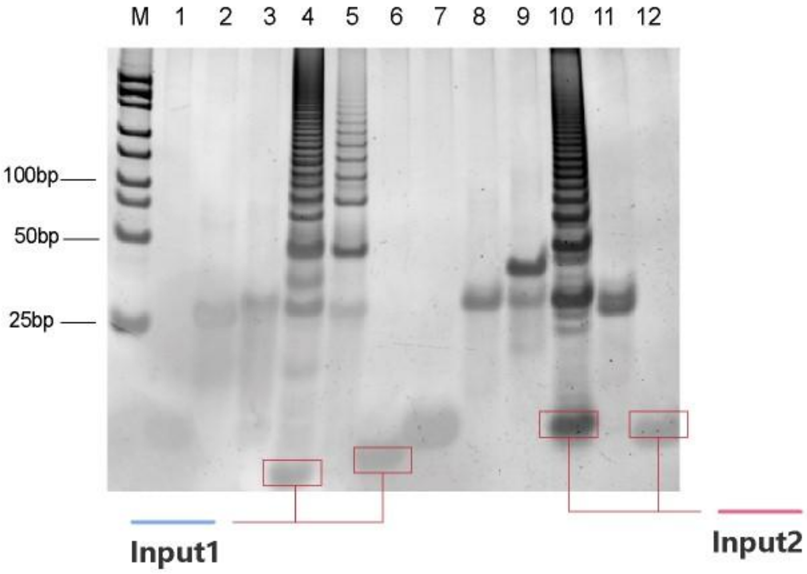

Figure S19 SDA characterization of AND / OR logic gate output signal. Lane 1 : Inititor-A. Lane 2 : AND / OR template A. Lane 3 : Inititor-A + AND / OR template A. Lane 4 : Amplification positive. Lane 5 : Amplification negative. Lane 6 : Input1 reference. Lane 7 : Inititor-B. Lane 8 : AND / OR template B. Lane 9 : Inititor-B + AND / OR template B. Lane 10 : Amplification positive. Lane 11 : amplification negative. Lane 12 : Input2 reference.

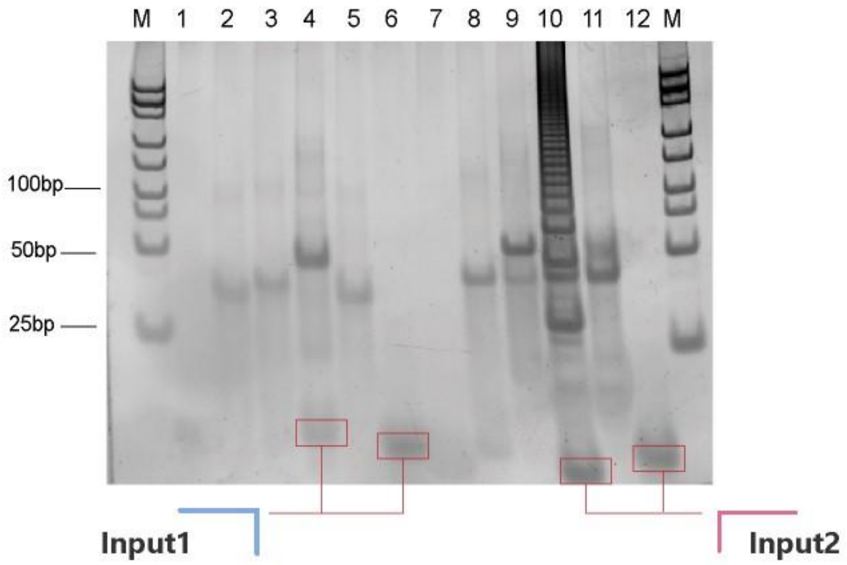

Figure S20 SDA characterization of NOR logic gate output signal. Lane 1 : Inititor-A. Lane 2 : NOR template A. Lane 3 : Inititor-A + NOR template A. Lane 4 : amplification positive. Lane 5 : amplification negative. Lane 6 : Input1 reference. Lane 7 : Inititor-B. Lane 8 : NOR template B. Lane 9 : Inititor-B + NOR template B. Lane 10 : amplification positive. Lane 11 : amplification negative. Lane 12 : Input2 reference

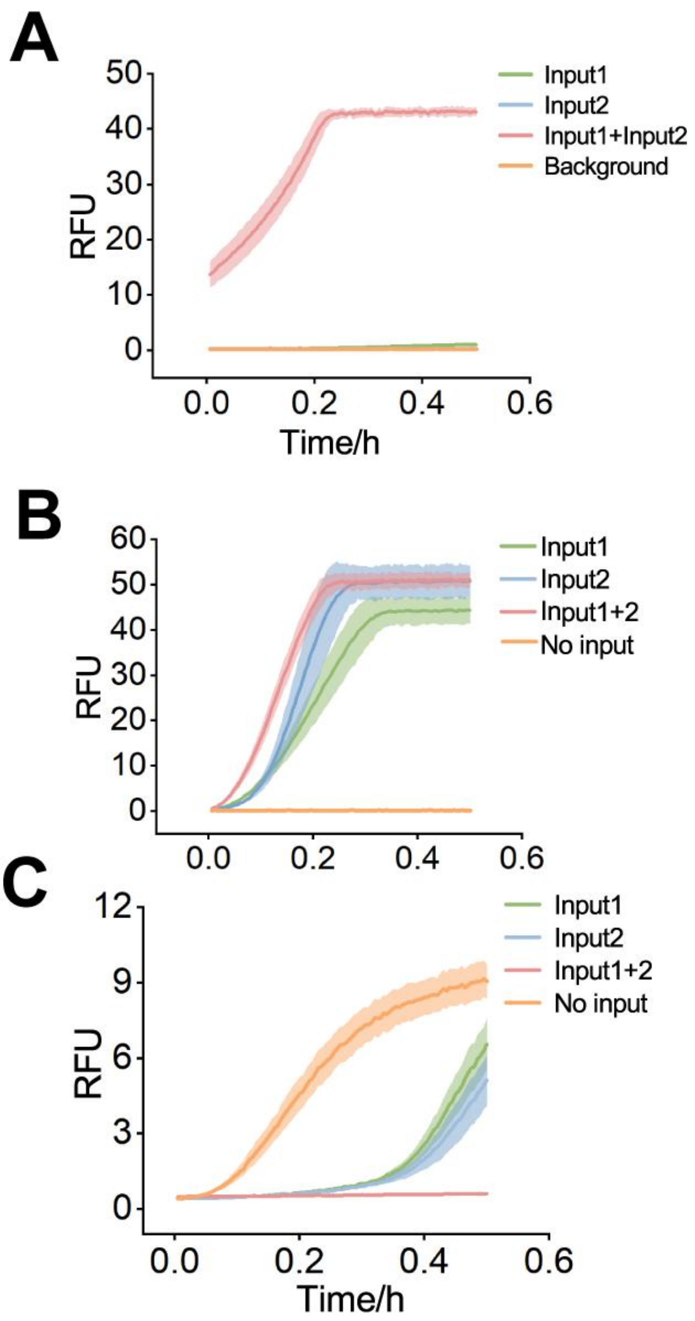

Figure S21 Verification of AND (A), OR (B), NOR (C) logic gates

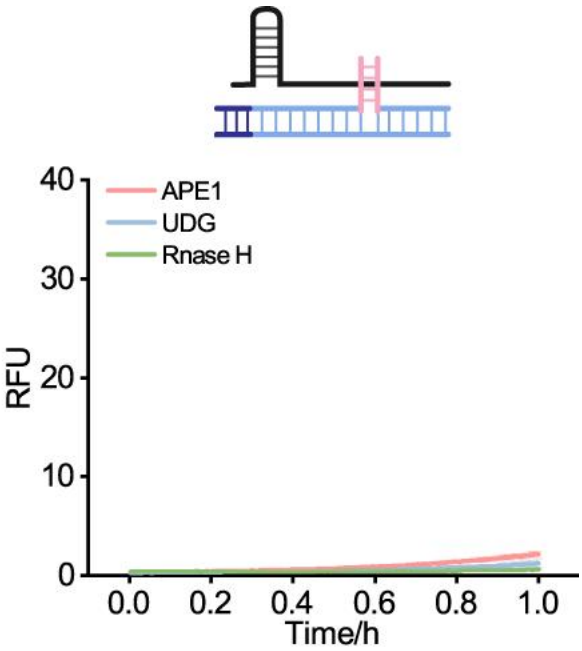

Figure S22 DsDNA with an 8 bp overhang cannot activate the trans-cleavage activity of Cas12a. (PAM lacking extra bases at the 3' end)

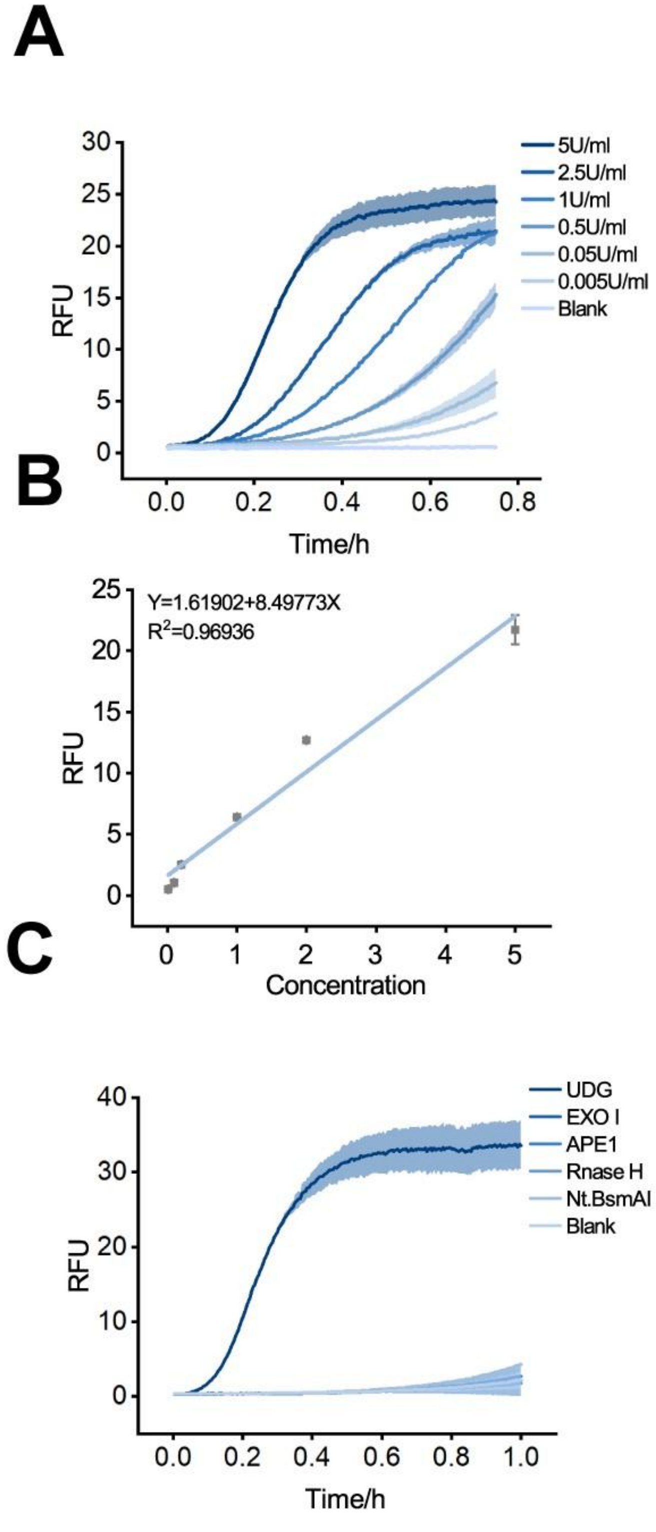

Figure S23 (A) Fluorescence plot of UDG detection at different concentrations. (B) The linear plot of UDG. (C) Specific detection of UDG.

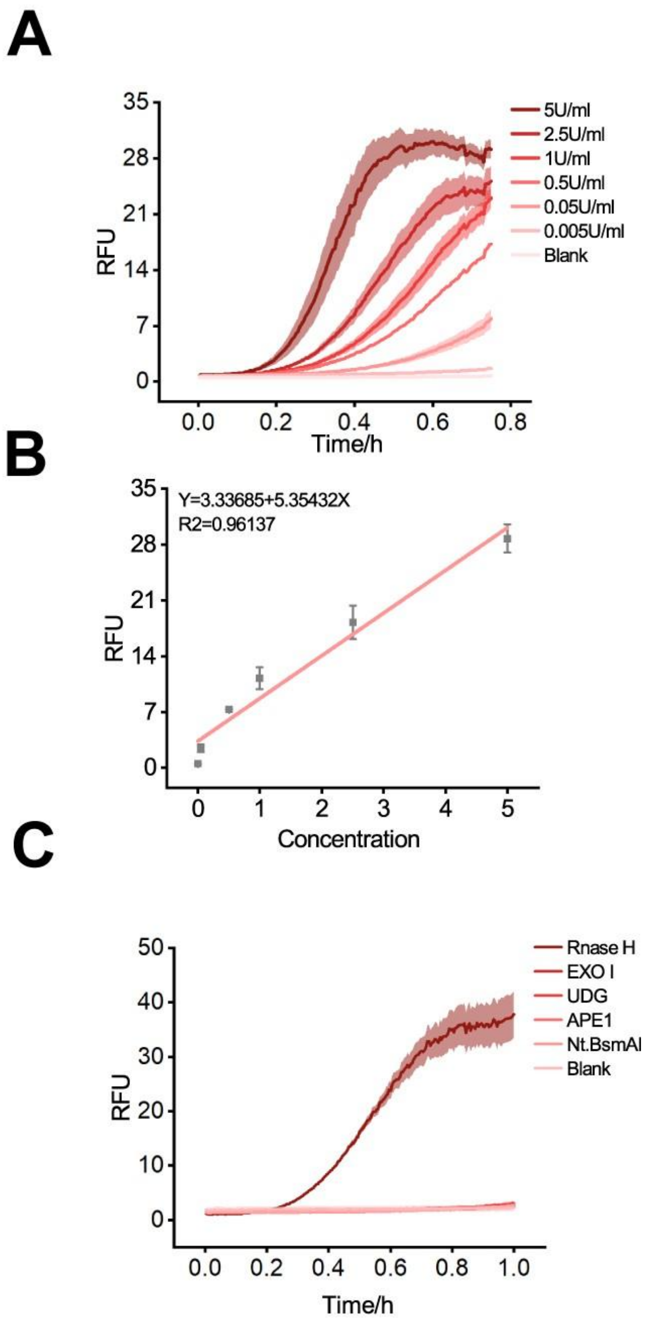

Figure S24 (A) Fluorescence plot of Rnase H detection at different concentrations. (B) The linear plot of Rnase H. (C) Specific detection of Rnase H.
